# Genetic variation regulates the activation and specificity of Restriction-Modification systems in *Neisseria gonorrhoeae*

**DOI:** 10.1038/s41598-019-51102-2

**Published:** 2019-10-11

**Authors:** Leonor Sánchez-Busó, Daniel Golparian, Julian Parkhill, Magnus Unemo, Simon R. Harris

**Affiliations:** 1 Centre for Genomic Pathogen Surveillance, Wellcome Sanger Institute, Wellcome Genome Campus, Hinxton, Cambridge UK; 2 Big Data Institute, Nuffield Department of Medicine, University of Oxford, Oxford, UK; 3 WHO Collaborating Centre for Gonorrhoea and other Sexually Transmitted Infections, National Reference Laboratory for Sexually Transmitted Infections, Department of Laboratory Medicine, Clinical Microbiology, Faculty of Medicine and Health, Örebro University, Örebro, Sweden; 4 Department of Veterinary Medicine, University of Cambridge, Cambridge, UK; 5 Microbiotica Ltd, Biodata Innovation Centre, Wellcome Genome Campus, Hinxton, Cambridge UK

**Keywords:** Bacterial genetics, High-throughput screening

## Abstract

Restriction-Modification systems (RMS) are one of the main mechanisms of defence against foreign DNA invasion and can have an important role in the regulation of gene expression. The obligate human pathogen *Neisseria gonorrhoeae* carries one of the highest loads of RMS in its genome; between 13 to 15 of the three main types. Previous work has described their organization in the reference genome FA1090 and has inferred the associated methylated motifs. Here, we studied the structure of RMS and target methylated motifs in 25 gonococcal strains sequenced with Single Molecule Real-Time (SMRT) technology, which provides data on DNA modification. The results showed a variable picture of active RMS in different strains, with phase variation switching the activity of Type III RMS, and both the activity and specificity of a Type I RMS. Interestingly, the Dam methylase was found in place of the NgoAXI endonuclease in two of the strains, despite being previously thought to be absent in the gonococcus. We also identified the real methylation target of NgoAXII as 5′-GCAGA-3′, different from that previously described. Results from this work give further insights into the diversity and dynamics of RMS and methylation patterns in *N. gonorrhoeae*.

## Introduction

*Neisseria gonorrhoeae* is a sexually-transmitted pathogen that causes gonorrhoea. Antimicrobial resistance in this pathogen is of great public health concern and there have recently been an increasing number of publications aimed at analysing its transmission and the evolution of genetic determinants of antimicrobial resistance using high throughput short-read sequencing^1^. However, Single Molecule Real-Time (SMRT) PacBio sequencing provides deeper information about microbial genomes, including data on DNA modification, mainly methylation in the form of 6mA, 5mC or 4mC. Several studies have focused on the reference strain *N. gonorrhoeae* FA1090 to characterize the population of Restriction-Modification systems (RMS) in the gonococcus^2–5^ and its methylation landscape^6^. However, RMS and methylation status are known to vary significantly between strains of the same species, so this provides only limited knowledge about RMS and methylation in the gonococcus as a whole.

*N. gonorrhoeae* is among the bacterial species with the highest numbers of RMS despite being naturally competent for DNA uptake^7^. Indeed, highly transformable bacteria have been found to contain a higher number of RMS compared with those which are less competent, acting as a defence system against the invasion of foreign DNA^8–10^. RMS are formed by a restriction endonuclease (REase) and a DNA methyltransferase (MTase) that recognize a specific pattern in the genome and act as multi-subunit complexes (Type I and III) or as independent enzymes (Type II)^10^. If the MTase is active, it will methylate the associated motifs and thus protect them from cleavage by the cognate REase. Bacteria can contain up to four types of RMS, although only Type I, II and III have been found in *Neisseria*^7,10^. Briefly, Type I RMS act as a multi-enzyme complex consisting of two MTase subunits (HsdM), two REase subunits (HsdR) and one specificity unit (HsdS) that recognizes specific asymmetric sequences, methylating both strands^10,11^. Type II RMS are generally formed by individual MTase and REase enzymes that recognize the same short palindromic motifs and perform a two-strand methylation or cleavage, respectively^10,11^. Finally, Type III RMS act as a complex of two Mod (MTase) and two Res (REase) subunits^12^ that recognize specific short asymmetrical motifs^10,11^. The specificity of Type I RMS is encoded by an independent gene (*hsdS*) that must be active for the other subunits to be functional, while Type III RMS harbour a DNA recognition domain (DRD) within the MTase^10^. RMS have also been associated with other roles apart from defending the genome against foreign DNA invasion, such as being involved in the epigenetic control of gene expression^8–10^. They have been described as selfish elements, tending to propagate on mobile genetic elements and promoting their own survival^13^. They are often associated with mobility genes, such as integrases or transposases and are sometimes flanked by repeats^14^. Indeed, they have been shown to have an important role in genetic flux among bacteria, as cleavage by REases provides fragments of double-stranded DNA that could be incorporated into the host genome^8,15^.

The action of some Type I and III RMS is known to be regulated by phase variation in several bacteria^7,10^. The variation in the number of repeat copies in homopolymers or short tandem repeats, mainly due to slipped-strand mispairing during DNA replication, can cause the switch between a functional, non-functional or a different version of the gene^9^. It has been hypothesized that phase variation can be used by RMS to regulate genome flux^8^ and it has been shown to be associated with a random switching in the expression of multiple genes (‘phasevarion’, for phase-variable regulon^10,16^) in *Haemophilus influenzae*^17,18^, *N. meningitidis*^19^, *N. gonorrhoeae*^2^, *Helicobacter pylori*^20^, and *Moraxella catarrhalis*^21^. Apart from the Type III RMS, the Type II ‘orphan’ Dam MTase has also been shown to be involved in gene regulation in several Gram-negative bacteria^9^ and is even required for virulence in some pathogenic organisms such as *Escherichia coli*, *Salmonella*, *Yersinia* or *Vibrio* species^22^. The Dam MTase participates in the methyl-directed DNA mismatch repair system and this constrains the flexibility of the bacteria to undergo phase variation, which involves mispairing of bases during replication slippage^23^. It has been found in some *N. lactamica* and *N. meningitidis* strains^24^, however, there have been no reports of *N. gonorrhoeae* carrying this enzyme until now. Instead, the gonococcus and other Dam-defective *Neisseria* contain a *dam replacing gene* (*drg*), characterized as an endonuclease^24^, that has been reported to be important for adhesion and biofilm formation^23^.

In this study, we characterised the population of RMS and their associated methylation specificities in 25 *N. gonorrhoeae* strains using SMRT PacBio data. Results from this study give a more comprehensive insight into the link between genomics and epigenomics in the gonococcus.

## Results

### Detection of RMS and associated DNA methylation

**Figure 1:**
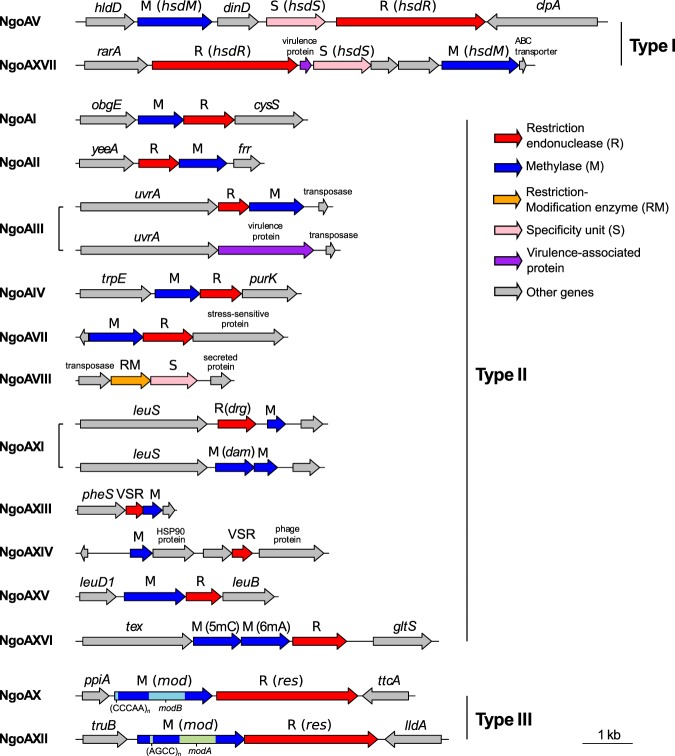
Genome organization of Restriction-Modification systems in *N. gonorrhoeae*. The nomenclature by Roberts *et al*.^11^ is shown between brackets for Type I and Type III RMS. The repeated unit of the phase-variable tandem repeat is shown for the *mod* genes as well as the alleles (*modA* and *modB*) of the DNA recognition domains (DRD). VSR: Very short patch repair endonuclease.

From 13 to 15 complete RMS were found in each of the 25 *N. gonorrhoeae* strains included in the study (Supplementary Table 1): 2 of Type I, 11 of Type II and 2 of Type III, all of which are present in the REBASE database (Fig. 1). A detailed scan of the genomes for genes with a Pfam annotation associated with a REase or a MTase did not detect any new RMS. Most of the systems were found surrounded by core genes coding for essential functions, such as tRNA aminoacylation during protein translation, ATP binding or magnesium ion binding, but also DNA transposition and isomerase activity (Supplementary Table 2). This could potentially be related to the fact that Type I REases have an important requirement of ATP for cleavage, and Type II REases require magnesium ion as a cofactor^11^. The amino acid translation of the repetitive region in the two Type III RMS is rich in prolines and, interestingly, the *ppiA* gene upstream of the methylase in NgoAX is an isomerase that catalyzes the isomerization of peptide bonds in prolyl residues, assisting protein folding^25^.

Analysis of DNA modification using PacBio sequencing for the 25 gonococcal strains produced a final list of 12 methylated motifs (Table 1, raw motif summaries in Supplementary Table 3). Manual curation of the raw list of motifs was performed by visualizing the distribution of interpulse duration (IPD) ratios for every base in each motif, which was essential to filter out artefacts produced by noise in the data. For example, some motifs were found to overlap with others methylated in a higher frequency or were inferred because of high IPD values in guanines, which can be caused by a O-6-methylguanine modification, associated with DNA damage by alkylating agents^26^.

**Table 1:** Restriction-Modifications systems (RMS) and target motifs in *N. gonorrhoeae*.

RMS type	RMS	Subtype (REBASE)	Motif	Methylation type	Detected?	PacBio/ REBASE	Methylated fraction F: % 5′-3′ R: % 3′-5′	Number of strains
I	NgoAV	Gamma	5′-GACN{6}TGC-3′ 3′-CTGN{6}ACG-5′	6mA	Yes	PacBio	F: 95.0-100 R: 91.3-99.6	2/25
			5′-GACN{7}TGC-3′ 3′-CTGN{7}ACG-5′	6mA	Yes	PacBio	F: 100 R: 100	4/25
			5′-GCAN{8}TGC-3′ 3′-CGTN{8}ACG-5′	6mA	Yes	PacBio	F: 99.8-99.9 R: 99.8-99.9	2/25
	NgoAXVII	Gamma	5′-GAGN{5}TAC-3′ 3′-CTCN{5}ATG-5′	6mA	Yes	PacBio	F: 100 R: 100	22/25
			5′-BGAGN{4}GTTAC-3′	6mA	Yes	PacBio	F: 87.5	1/25
II	NgoAI	P	5′-RGCGCY-3′ 3′-YCGCGR-5′	5mC	Yes	REBASE	—	25/25
	NgoAII	P	5′-GGCC-3′ 3′-CCGG-5′	5mC	Yes	REBASE	—	25/25
	NgoAIII	P	5′-CCGCGG-3′ 3′-GGCGCC-5′	4mC	Yes	PacBio	50.4	21/25
	NgoAIV	P	5′-GCCGGC-3′ 3′-CGGCCG-5′	5mC	Yes	REBASE	—	25/25
	NgoAVII	S	5′-GCGGC-3' 3′-CGCCG-5′	5mC	Yes	PacBio	36.3–39.7	25/25
	NgoAVIII	G,S	5′-GACN{5}TGA-3′ 3′-CTGN{5}ACT-5′	6mA	No	REBASE (base undetermined)	—	—
	NgoAXI (Dam)	Beta	5′-GATC-3′ 3′-CTAG-5′	6mA	Yes	PacBio	F: 98.6–99.0 R: 98.6–99.0	2/25
	NgoAXIII	—	Unknown	Unknown	No	REBASE	—	—
	NgoAXIV	P	5′-CCGG-3′ 3'-GGCC-5′	5mC	Yes	REBASE	—	25/25
	NgoAXV	P	5′-GGNNCC-3′ 3'-CCNNGG-5′	5mC	Yes	REBASE	—	25/25
	NgoAXVI	S	5′-GGTGA-3′ 3′-CCACT-5′	Forward 6mA Reverse 5mC	Yes	PacBio	F: 93.1–99.9	25/25
	Unknown	Unknown	5′-AAANCGGTTNNC-3′ 3′-TTTNGCCAANNG-5′	m4C	Yes	PacBio	F: 17.5–20.6	>14/25
III	NgoAX	—	5′-CCACC-3′	6mA	Yes	PacBio	F: 97.3–100	6/25
	NgoAXII	—	5′-GCAGA-3′	6mA	Yes	PacBio	F: 95.4–98.2	3/25

Those detected as methylated by the PacBio SMRT pipeline are marked as such and the fraction of modified motifs in the genomes indicated as a range (F: forward, R: reverse). For those not directly detected by the pipeline, the associated motifs in REBASE are shown and marked as detected if a significant difference in IPD ratios is found between the target base and the unmethylated equivalent by a Mann-Whitney test.

When looking at raw IPD ratios, it is difficult to set a threshold to distinguish between methylated and unmethylated bases. To confirm they can be differentiated, we investigated the distributions of IPD ratios for methylated and unmethylated bases. As expected, 6mA modifications extracted from our final motif list showed the highest IPD ratio values, with a mean value of 5.63 (Supplementary Fig. 1). Cytosine methylations (4mC and 5mC) are difficult to detect using native DNA, especially 5mC, because of the lower effect on delaying the polymerase during PacBio sequencing. Even so, they showed an average IPD ratio of 3.24 and 2.81, respectively, higher than the mean ratios for any unmethylated base (Supplementary Fig. 1). These results confirm that there is enough differentiation between methylated and unmethylated bases to draw further conclusions about methylated regions in the genome.

### Type I RMS: NgoAV is multispecific

**Figure 2:**
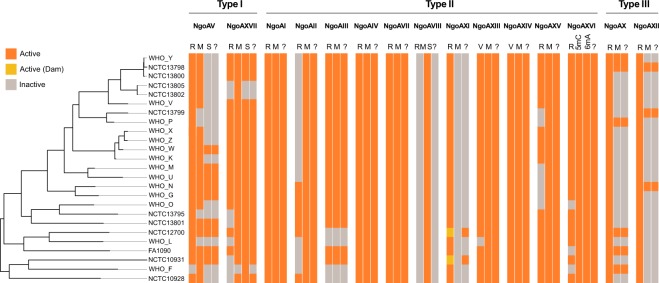
Active and inactive Restriction-Modification systems (RMS) in the 25 *N. gonorrhoeae* strains under study. The Dam methylase is marked in a different colour (see legend). An extra column (‘?’) in each RMS is plotted showing if methylation is expected (orange) for each strain. The tree on the left is a maximum likelihood reconstruction of the core genome SNPs of the 25 strains.

**Figure 3:**
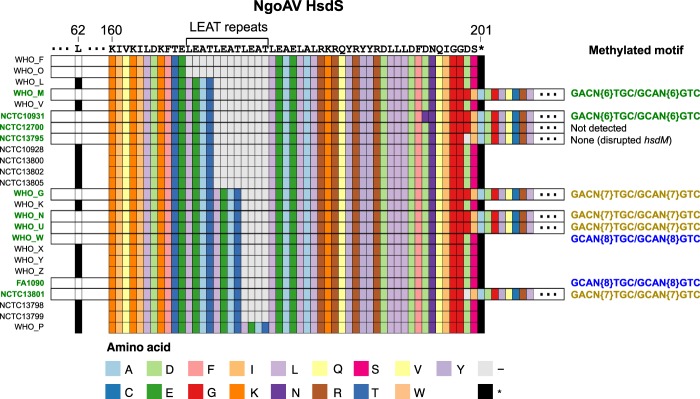
Protein alignment of the HsdS specificity unit of the Type I NgoAV Restriction-Modification system (RMS). The three main regions of variation within the unit are shown as a premature stop codon in position 62 of the protein that causes inactivation of the RMS, a variable number of LEAT repeats and a downstream stop codon in position 201 (caused by a change in the length of a poly-G homopolymer) which, in combination, are related to a different recognition motif. The strains with a full-length protein are labelled in green (left), and the methylated motifs detected using PacBio are shown in different colours on the right. ‘−‘ represents gaps and ‘*’ stop codons.

Two Type I RMS were present in the *N. gonorrhoeae* strains (Figures 1 and 2). Type I NgoAV RMS showed at least three different regions of genetic variability within the specificity unit (*hsdS*). An insertion of a T at position 186 of the gene creates a frameshift that causes a premature stop codon in position 62 of the protein, inactivating *hsdS*, and thus, the whole RMS (Fig. 3). From 0 to 3 repeats of the amino acid sequence LEAT^27^ were observed in different strains. Some strains also contained a downstream frameshift caused by the deletion of two Gs in a homopolymer, alterations of which have previously been associated with a change of NgoAV specificity^4^. This second frameshift does not cause the inactivation of the gene, but in combination with the number of LEAT repeats, was found to be associated with a change in the recognition pattern (Fig. 3 and Supplementary Table 4).

Only one methylation target is currently shown for NgoAV in REBASE (5′-GCAN{8}TGC-3′). However, we also observed two other methylation targets associated with this RMS. Two strains with 1 LEAT repeat and no frameshift showed two-strand 6mA methylation in the 5′-GACN{6}TGC-3′ motif (Supplementary Table 4 and Supplementary Fig. 2). However, four strains with 2 LEAT repeats instead showed the same starting and ending triplet nucleotides in the recognition pattern but the spacer was 1 bp longer, 5′-GACN{7}TGC-3′ (Supplementary Table 4 and Supplementary Fig. 3). Finally, two strains showed 5′-GCAN{8}TGC-3′ methylation, slightly different in the 5′ end and a further 1 bp longer (Supplementary Table 4 and Supplementary Fig. 4). In this case, they contained 2 LEAT repeats and the frameshift downstream that causes a premature end of the protein, which does not inactivate it but changes its specificity (Fig. 3). The two motifs not annotated in REBASE to NgoAV were assigned to this RMS because they are double-stranded methylated asymmetric motifs and the methylation signal was coherent with the pattern of variability within the NgoAV *hsdS*. The only exception was strain NCTC12700, which contained complete sequences of the *hsdM* and *hsdS* genes, which should translate to full-length proteins. HsdS contains 1 LEAT repeat and no middle frameshift as with WHO M or NCTC10931, but no methylation of the corresponding motif (5′-GACN{6}TGC-3′) is found from the PacBio data (Supplementary Table 4). This could be caused by any of these proteins not being translated due to genetic regulation or posttranslational modifications impeding its action.

The second Type I RMS, NgoAXVII, did not show particular variable regions in the specificity unit or methylase among the strains, as happens with NgoAV *hsdS*, but only the disruption of one and/or the other was found to cause the inactivation of the whole RMS. All strains with full-length HsdM and HsdS subunits in this RMS showed two-strand 6mA methylation in the 5′-GAGN{5}TAC-3′ motif (Supplementary Fig. 5 and Supplementary Table 5). An additional motif was also detected in WHO O that we hypothesized is an off-target methylation of this enzyme because of the similarity of the motifs: 5′-BGAGN{4}GTTAC-3′, very similar to the main motif methylated by NgoAXVII but one base longer (Table 1). The specificity unit of this enzyme was found to be very conserved, with only two non-synonymous mutations that had no association with the potential off-target methylations.

### Cytosine methylation by Type II RMS

*N. gonorrhoeae* contains up to 11 Type II RMS (Figures 1 and 2), 7 of them annotated as associated with cytosine methylation (mC) in REBASE. In spite of apparently showing functional cytosine methylases, only three motifs containing 5mC or 4mC methylation were detected by the PacBio pipeline (Table 1 and Supplementary Table 3). However, as we show above, both types of cytosine modification can be distinguished from an unmethylated base (Supplementary Fig. 1). In order to perform a deeper analysis of the motifs associated with mC in Type II RMS, we specifically targeted those reported in REBASE and evaluated their per-base IPD ratios (Supplementary Table 6). A Mann-Whitney test was performed between the distribution of IPD values for the predicted mCs and a random set of unmethylated cytosines for each strain to assess whether there was a statistically significant difference. Significant cytosine modification was found for the motifs associated with the 7 RMS expressing a functional methylase (Supplementary Fig. 6 and Supplementary Table 6). Four strains showed a gene annotated as “virulence-associated” instead of the NgoAIII RMS and three of those did not show significant signals of methylation (Supplementary Table 6). The motif associated with NgoAIII in REBASE is 5′-GGCGCC-3′ (5mC) and this was detected as a 4mC signal (reported as the degenerate motif 5′-GGCSCCND-3′) in WHO V (Supplementary Table 3), although we showed the signal was in fact present in all the strains with a functional RMS (Supplementary Fig. 6). The WHO G PacBio data produced a non-significant test for mC in the forward strand of 5′-GCGGC-3′, associated with NgoAVII, which could be due to hemi-methylation or poor signal in the data.

RMS NgoAXIII was found as a VSR (Very Short patch Repair) protein followed by a MTase and there is no report of its target motif in REBASE. A previous study experimentally confirmed that this MTase is inactive in FA1090^28^. A series of BLAST searches revealed that only *N. polysaccharea* M18661 (Genbank accession number CP031325.1) contained a homologous region to NgoAXIII. Instead, genomes of *N. lactamica* (i.e. Y92-1009, Genbank accession number CP019894.1) showed a longer version of the MTase (362 amino acids compared to 112 in *N. gonorrhoeae* FA1090 and 133 in the rest). This shows that *N. gonorrhoeae* does not have a complete NgoAXIII system, but only remnants of a MTase. Interestingly, several *N. meningitidis* genomes contain the VSR protein followed by a different RMS instead (NmeDI)^28^.

The 5′-AAANCGGTTNNC-3′ motif was detected by the PacBio SMRT pipeline containing single-strand m4C methylation in the underlined base. The study of the distribution of per-base IPD ratios in the WHO strains revealed that the complementary sequence was probably also methylated at 3′-TTTNGCCAANNG-5′ (Supplementary Fig. 7). A Mann-Whitney test revealed the distribution of IPD values for these two bases to be significantly different than that of an unmethylated cytosine for most of the strains (Supplementary Fig. 8). FA1090 and, especially, the NCTC strains, consistently showed very noisy results, but the test was still statistically significant for them (Supplementary Fig. 8). We hypothesize that the methylation of this motif could result from a secondary target of a Type II RMS although this is unlikely as the fraction of methylated 5′-AAANCGGTTNNC-3′ is very low (Table 1).

### 6mA methylation by Type II RMS

Three of the Type II RMS contained 6mA methylases. The NgoAVIII system has a BcgI-like structure^29^ as it is formed by an enzyme with restriction and 6mA methylation properties (RM) and a specificity unit (S). A BLASTn search of the genomic region spanning both units against the NCBI database revealed that this RMS has not been described in any other organism. A transposase immediately upstream of the RM unit indicates that this system may be mobile and acquired from an unknown source. The target motif in REBASE for this RMS is that of BcgI, 5′-GACN{5}TGA-3′, although we do not detect this motif as methylated or any other motif with 6mA methylation that could be associated with NgoAVIII (Supplementary Table 3), despite the system appearing intact. Interestingly, BcgI-like MTases are known to prefer hemi-methylated DNA, although their recognition sequences are very similar to those of Type I MTases, which methylate both strands^30,31^. Thus, NgoAVIII may have a maintenance role for the motifs targeted by Type I MTases, methylating the growing strand immediately after chromosomal replication, a point when those motifs will be hemi-methylated.

The Dam methylase was found in place of the restriction enzyme in NgoAXI in two strains (NCTC10931 and NCTC12700), which showed double-strand 6mA 5′-GATC-3′ methylation (Supplementary Fig. 9). This MTase has been reported to be substituted by the Dam-replacing endonuclease (Drg) in many *Neisseria* species, but has not been found in *N. gonorrhoeae* until now^23,24^. Finally, the NgoAXVI RMS is formed by two methylases with 5mC and 6mA methylation specificities, respectively, and a restriction enzyme. A clear 6mA signal is found in the associated 5′-GGTGA-3′ motif in all the strains (Supplementary Fig. 10). A more detailed comparative analysis of the distribution of IPD values of the three cytosines in the reverse complement of this motif against unmethylated Cs for each strain identified the middle cytosine as the most plausible candidate for 5mC methylation (3′-CCACT-5′ Supplementary Fig. 11).

### Type III RMS: the target of NgoAXII is 5′-GCAGA-3′

Two Type III RMS were present in the *N. gonorrhoeae* strains (Figures 1 and 2). These contain a phase-variable methylase (Mod) that controls the activation or inactivation of the system and includes a DNA-recognition domain (DRD) that controls its specificity. NgoAX^3^ contained a variable number of 5′-CCCAA-3′ repeats that switch the system ON/OFF and the *modB1* DRD allele (Supplementary Table 7). In contrast, NgoAXII contained a variable number of 5′-AGCC-3′ repeats and the *modA13* DRD allele (Supplementary Table 8). Both *modA* and *modB* DRD alleles are those typically found in *N. gonorrhoeae*^5,32^.

**Figure 4:**
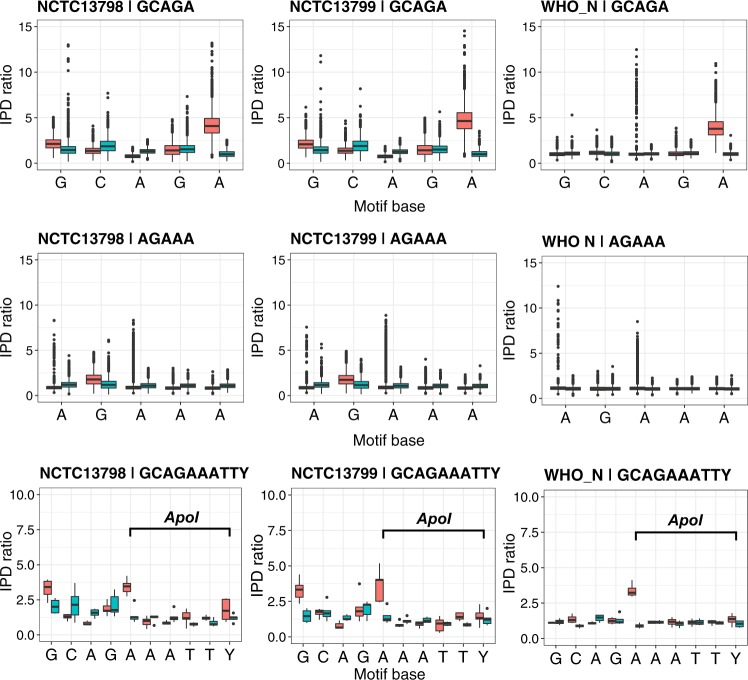
Comparison of the per-base distribution of IPD ratios for the 5′-GCAGA-3′ motif detected in three strains as associated with NgoAXII, the distributions for the previously inferred 5′-AGAAA-3′ and the motif overlapping between the previous two and the recognition pattern of the ApoI restriction enzyme, 5′-RAATTY-3′^2^ in the three strains with a functional NgoAXII system. Two boxplots are shown per base, corresponding to the IPD ratios of the forward (red) and reverse (blue) strands.

Active NgoAX RMS showed 6mA 5′-CCACC-3′ methylation in 6 strains (Supplementary Fig. 12 and Supplementary Table 7), and active NgoAXII showed 6mA 5′-GCAGA-3′ methylation in 3 strains (Supplementary Fig. 13 and Supplementary Table 8). A previous report suggested the methylated motif for the *modA13* allele of this enzyme was 5′-AGAAA-3′ in strain FA1090 by evaluating the digestion pattern of ApoI and other enzymes^2^. This is the recognition motif associated with NgoAXII in REBASE, although it has not been detected in PacBio studies^6^. The sequence of the DRD region was identical among the three strains and FA1090, providing further evidence that there are no mutations associated that could explain a different specificity among FA1090 and the three strains in this study. However, our analysis of the distribution of the per-base IPD ratios for both motifs in the three strains with an active *modA* (Fig. 4) revealed that less than 0.1% of the AGAAA motifs contained an IPD ratio above 3, while most of the GCAGA (>79%) showed high IPDs in the underlined base (Supplementary Table 9). We only observed between 4 and 8 motifs in the genomes that included both motifs overlapping with each other and that expanded to include a complete ApoI recognition sequence (5′-RAATTY-3′), 5′-GCAG**A**AATTY-3′. Therefore, the digestion in these cases could have been impeded by the methylation of the second A instead (in bold). A low fraction of motifs with high IPDs in the first A of 5′-GCAGA-3′ was found in the strains with an active Type I NgoAV, and this may be due to overlapping signals between both RMS (Supplementary Figures 2–4 and 13).

Strangely, results from the motif analysis in FA1090 did not show signals of 5′-GCAGA-3′ methylation, although the *mod* gene was apparently complete in the reference genome (NCBI:AE004969). An assembly of the PacBio reads from which the methylation data was obtained for this strain^6^ revealed that *mod* in the NgoAXII RM system in the subclone of FA1090 used for sequencing in the cited work contained 35 AGCC repeats instead of 37 in the reference genome, causing a protein truncation and thus a lack of 5′-GCAGA-3′ methylation. This difference in the number of repeats was caused by phase variation probably during subculturing, and inactivation of the *mod* gene has been associated with a change in gene expression^17^.

### Orphan methylases in *N. gonorrhoeae* include the Dam methylase

Disrupted methylases can cause the inactivation of the whole RMS due to mechanisms that avoid self-degradation of the chromosomal DNA^10^ in this situation. However, methylases can be functional with a disrupted or absent endonuclease. These methylases have been associated with a regulatory rather than defensive role and have been described as ‘orphan’. In the strains under study we observed multiple examples of this in the Type I NgoAXVII (Supplementary Table 5) and Type II NgoAII RMS (Supplementary Table 6). The Dam methylase in NgoAXI acts as an ‘orphan’ methylase, as it completely replaces the endonuclease of the system, and the downstream MTase is disrupted. A BLASTp search at the NCBI non-redundant protein database revealed that the gonococcal Dam sequence has 97–98% amino acid identity to *N. polysaccharea*, 97% to *N. flavescens*, 96–97% to *N. lactamica*, and 92–96% to *N. meningitidis*. A broader screening of the Dam protein in the *Neisseria* genus showed a reasonable amount of diversity at the amino acid level, with recombination swapping sequence variants among *Neisseria* species (Supplementary Fig. 14).

A scan of the Pfam domains of the proteome of the 25 *N. gonorrhoeae* strains did not reveal further DNA methylases without an accompanying restriction enzyme in the main chromosomes. However, the strains carrying the conjugative plasmid (WHO G, L, M, N, O, W, and NCTC10931) showed an orphan MTase in this element that has a 100% amino acid and nucleotide identity to the Type II M.Ngo8107ORF11P and M.Ngo5289ORFAP enzymes in REBASE (Supplementary Fig. 15). No further matches were found to any other bacteria in a BLASTn search against a non-redundant nucleotide database.

## Discussion

Restriction-Modification systems have been shown to perform several other roles apart from being a defence system against foreign DNA^8^. For example, some of them, especially those from Type III, are involved in the control of gene expression through phase variable repeats in the Mod unit^16^. Previous papers have characterised different RMS mostly in the *N. gonorrhoeae* FA1090 reference strain^2–5,33^ and also data on its methylation status has been described^6^. In this manuscript, we provide a detailed view of the structure and variability of DNA MTases and specificity units in RMS in 25 strains of *N. gonorrhoeae* and link them to the detected methylated motifs.

The analysed gonococcal genomes showed from 13 to 15 complete RMS, all of them described in the reference strain FA1090 in REBASE^34^. Twelve motifs were detected as methylated in the 25 strains, most of them containing 6mA methylation (Table 1). In the case of the Type I NgoAV, earlier work confirmed the functionality of the naturally truncated *hsdS* in FA1090, which was predicted to recognize the 5′-GCAN{8}TGC-3′ motif^35^. Later, it was proven that the cause of this truncation was a phase-variable G homopolymer and that the restitution of a complete form of this unit changed the specificity of the MTase to 5′-GCAN{7}STGC-3′^4^. Our study reveals further insight into the diversity of specificities of the NgoAV RMS as two other different motifs (5′-GACN{6}TGC-3′ and 5′-GACN{7}TGC-3′) were detected in strains with a functional RMS. The change in specificity was found to be related to a combination of two factors: the number of LEAT repeats in the protein sequence of *hsdS* and the presence of the downstream frameshift that causes the previously-described truncation^4^ (Fig. 3 and Supplementary Table 4). The presence of amino acid repeats in the centre region of HsdS and its impact on specificity was first described in *Escherichia coli* (TAEL repeats)^36^ and has also been described in previous works in *N. gonorrhoeae* (LEAT or EATL repeats)^27,35^, but not its role in sequence specificity in combination with the downstream frameshift. Off-target methylated motifs were associated with the other Type I RMS NgoAXVII, although no associated variability was found in *hsdS* in this system.

Seven out of 11 Type II RMS in *N. gonorrhoeae* are associated with cytosine methylation. However, this type of DNA modification is difficult to detect by current PacBio sequencing, and high coverage in combination with Tet-conversion is preferable^37^. Only three motifs associated with 5mC or 4mC methylation were detected in a subset of the strains (Table 1). Nonetheless, a statistical comparison between the IPD values of target cytosines in the motifs annotated in REBASE for these RMS to those from unmethylated cytosines revealed significantly higher values for those in the strains carrying active 5mC MTases (Supplementary Table 5). 5mC can be converted into T by deamination, generating mismatches that can produce mutations. Bacteria can modulate this effect by methylating 4mC instead^8^. In fact, double-stranded methylation of 5′-CCGCGG-3′ by NgoAIII was detected by the PacBio system as 4mC, apart from the potential off-target 5′-AAANCGGTTNNC-3′ motif (Table 1). Interestingly, secondary methylation by these types of enzymes has been observed in *H. influenzae*^38^.

*N. gonorrhoeae* harbours two VSR endonucleases to correct T:G mismatches, V.NgoAXIII and V.NgoAXIV^33^. In this study, we observed a truncated NgoAXIII MTase in all strains, as previously described for FA1090^28^. However, the VSR may still be functional, as it has been found to recognize T:G mismatches in every nucleotide context^33^. V.NgoAXIV has also been described to recognize mismatches in sequences other than 5′-CCGG-3′^33^. No methylated motif has been found for the Bcg-like RMS NgoAVIII, however, previous reports describe this type of MTase as having a strict preference for hemi-methylated sites^30,31^ in Type I-like motifs. Thus, we propose that NgoAVIII may have a maintenance role, methylating the replicated strand in sites targeted by Type I RMS during DNA replication.

The Dam methylase is present in some strains of *N. lactamica* and *N. meningitidis*^24^. However, it has not been described in *N. gonorrhoeae* until now. Instead, *Neisseria* species or strains lacking this enzyme are known to carry an endonuclease encoded by *drg* (*dam* replacing gene) recognizing the 5′-GATC-3′ motif^24,39^. Here, we observed two *N. gonorrhoeae* strains (NCTC10931 and NCTC12700) carrying the *dam* MTase gene instead of the *drg* gene in the NgoAXI RMS locus, followed by a truncated MTase. Thus, Dam is acting as an orphan enzyme, a form that has also been shown to regulate gene expression^40^. Interestingly, previous publications have suggested that strains carrying the *drg* gene have an advantage over those carrying *dam* because they have more flexibility for phase-variation, as the Dam MTase participates in DNA mismatch repair during replication^23,39^. Also, the same publications experimentally showed that strains carrying *drg* form more stable biofilms and have better adhesion to human cells during infection.

Several works have studied the ‘phasevarion’ in *N. gonorrhoeae* and *N. meningitidis*, referred to the set of genes with an altered expression due to phase variation in a MTase^2,10,17^. Type I NgoAV has been considered to regulate a phasevarion as the phase variation of the G homopolymer is involved in a change in specificity^4,16^. In our work, we complement this result, as we show that the change in specificity is produced as a combination of the phase-variable poly-G and the number of LEAT repeats^27^. However, the best examples of phasevarion are the phase-variable Type III RMS^10^, in which for example *modD1* has been associated with hypervirulent *N. meningitidis* clonal complexes^19^ and *modA13* with enhanced biofilm formation and intracellular survival^16^. In a recent publication, the NgoAX Mod was experimentally inactivated in *N. gonorrhoeae* and a deregulation of the expression of 121 genes was observed, along with an effect on the adherence to and invasion of host epithelial cells, which are essential for infection^5^. In contrast, inactivation of the NgoAXII Mod enzyme showed a deregulation of 54 genes under iron-limiting conditions^2^. The *N. gonorrhoeae* population has been reported to carry a very conserved *modB1* allele compared to *modA13*, which has led to the suggestion that NgoAX Mod is the main regulator of the epigenome, while NgoAXII regulates in specific growth conditions^5^. Here, we observe that indeed all the strains under study carry the *modA13* and *modB1* alleles, with all possible combinations of activity apparent: one of the two Mod active (methylates the target motif), both active or both inactive (Supplementary Table 7 and Supplementary Table 8). In this work we show that the real methylation target of NgoAXII is 5′-GCAGA-3′, different from that annotated in REBASE for this RMS, highlighting the necessity of using experimental data such as PacBio kinetic information, to update this and other databases.

Results from this study shed further light onto the path to understanding RMS and the role of methylation in bacteria, and particularly in *N. gonorrhoeae*. However, we want to emphasize that the activation or inactivation of many of the RMS are produced in a reversible manner through phase variation. This means that different culture conditions or growth phases of the same strain can have a different picture of functional RMS. We have indeed observed this in the present work with the inactivation of the NgoAXII Mod unit in the subclone of FA1090 used to produce the PacBio data used in this study^6^. It is also important to mention the limitations of high throughput sequencing approaches, which can struggle to report the real length of a repeat, especially if there is a subpopulation with different repeat lengths and a consensus sequence is given. Further studies on larger collections of *N. gonorrhoeae*, different culture conditions and growth phases will allow a better understanding of when RMS are activated and the proportions of active RMS in the gonococcal population.

In summary, genetic diversity created through phase variation or hypervariable domains controls the activation or inactivation of RMS in *N. gonorrhoeae*, along with a variation of specificity in the Type I NgoAV RMS. A change in the number of tandem repeats and homopolymers through phase variation can happen in a reversible manner to control the ON/OFF switching of an RMS according to the selection acting on the bacteria, i.e. defence against a particular stress, immune evasion, etc. This work gives further insight into the RMS in *N. gonorrhoeae* and the consequent methylation landscape, which can change to modulate gene expression. We also show the importance of detailed PacBio SMRT analysis to enhance and complete the methylation information contained in public databases.

## Methods

### Genomes included in the study

We analysed the genomes of a total of 25 *N. gonorrhoeae* strains sequenced using PacBio. Of those, 14 complete genomes were obtained from the 2016 WHO reference panel^41^ and 10 additional genomes were selected from the Public Health England NCTC 3000 collection (http://www.sanger.ac.uk/resources/downloads/bacteria/nctc/) avoiding overlap with the WHO panel, to further improve the representativeness of the *N. gonorrhoeae* genome diversity (Supplementary Table 1). Additionally, PacBio raw data and predicted motifs were retrieved for the reference genome *N. gonorrhoeae* FA1090 from Blow *et al*.^6^. Sequencing was run using native DNA in all cases. Assembly and annotation were performed as reported in the publications cited above, using an automatic and improved pipeline at the Wellcome Sanger Institute^41^. FA1090 was reassembled using the PacBio data with Canu v1.6^42^ to compare the number of tandem repeats in the Type III MTases with those from the original assembly available in the public databases (GenBank accession AE004969).

### Analysis of DNA methylation

Genome-wide base modifications and predicted modified motifs were called using the RS_Modification_and_Motif_Analysis protocol from the PacBio SMRT Analysis software v2.3.0. Coordinates of the predicted motifs were localized in all the genomes and plasmids using the EMBOSS application *fuzznuc*^43^. Per-base IPD ratios for the predicted motifs were extracted from the raw data and visualized in the 25 strains using R^44^.

The distribution of IPD values for each of the four bases outside any of the predicted motifs was used as the distribution of values for the four unmethylated bases. A random subsample of 10,000 unmethylated sites of each base was used. Cytosine methylation from Type II RMS was inferred by evaluating the distribution of IPD ratios in the target base in the associated motif in REBASE^34^ compared to that of unmethylated cytosines. A Mann-Whitney test was performed to evaluate statistical significance and p-values were corrected using Bonferroni^45^.

### Detection and specificity of RMS

An extended and manually curated list of Pfam domains associated with REases and MTases from a previous work^46^ was used to detect these genes in the annotations of the genomes under study. HMMER^47^ *hmmscan* was run independently on the proteome of each genome (.faa files) against Pfam(A) v30^48^ to complement the annotation information. R language^44^ was used to parse the annotation files and the results from *hmmscan* and extract genes with an inferred Pfam domain in the list of target REases and MTases. Genes predicted to be an REase or an MTase that were less than 10 genes distant were considered to be part of the same RMS. The rest were tagged as ‘orphan’. Results were compared to the RMS annotated for FA1090 in REBASE^34^. The presence or absence of every RMS in each strain was visualized using *phandango*^49^.

The motif annotated in REBASE^34^ for each MTase was compared to the list of predicted motifs obtained by the PacBio SMRT Analysis software for all the strains. In REBASE, many methylation targets are inferred across RMS that have similar amino acid sequences. However, a small change in the sequence of the DNA recognition domain of a methylase or the specificity unit among species with orthologous RMS can alter the sequence that is methylated. This means that matching the methylated motifs obtained by PacBio with the annotated methylases in REBASE is sometimes not straightforward and new methylation signals can be found that are associated with existing annotated methylases. These cases were further investigated and assigned to a RMS using the genomic data and according to the characteristics of the newly-found methylated motifs (one/two-strand methylation, palindromic/asymmetric motif, length, etc)^10^. Nucleotide and protein sequences of every unit of the RMS were extracted for each strain and compared using SeaView v4.6.1^50^ to look for sources of variability within the MTases or the specificity units. These and the flanking genes were visualized using Artemis v16.0.0^51^. Disrupted proteins were confirmed by a protein-protein BLAST against a non-redundant protein database^52^.

Representative protein sequences of the Dam methylase in the *Neisseria* genus were downloaded from the Identical Protein Groups (IPG) tool of the NCBI database (accessed on 21/01/2019). These sequences were aligned using SeaView v4.6.1^50^ and the resulting alignment trimmed using Gblocks v0.91b^53^ considering only positions in which at least half of the sequences do not have a gap. PhyML^54^ was used to build a maximum likelihood tree using the LG model and performing 100 bootstrap replicates. Final tree and metadata were visualised using iToL^55^.

### GO enrichment analysis

A Gene Ontology (GO) enrichment analysis was performed for the genes flanking all the RMS using the *topGO* R package^56^. The three ontologies were scanned (‘BP’, biological process; ‘MF’, molecular function; and ‘CC’, cellular component). The *classic* and *weight01* algorithms were used which do not, and do, use hierarchy information for scoring a particular GO term, respectively^57^. The statistical significance of the enrichment was calculated using a Fisher’s exact test between the observed and expected number of genes in each term for each algorithm. Terms with a p-value < 0.05 in both algorithms were considered as significant in the results. P-values were not corrected for multiple testing to avoid excluding significant GO terms near the cut-off. Besides, the tests are not independent when using the *weight01* algorithm as it is conditioned on neighbouring terms^56^.

## Supplementary information

Supplementary Information

Supplementary Table 3

## Data Availability

Scripts used to perform the analyses and plots in this work are available in the GitHub repository https://github.com/leosanbu/MethylationProject. The 14 *N. gonorrhoeae* PacBio raw data and complete genomes from the 2016 WHO panel are available under the ENA Bioproject PRJEB14020 (Sample accessions SAMEA2448460-SAMEA2448470 and SAMEA2796326-SAMEA2796328)^41^. Accession numbers for the PacBio data from the 10 strains downloaded from the NCTC3000 project are available in the following link: https://www.sanger.ac.uk/resources/downloads/bacteria/nctc/ (Sample accessions SAMEA3174297-SAMEA3174299, SAMEA4076737, SAMEA4076741, SAMEA4076765, SAMEA4076768-SAMEA4076770, SAMEA4076773). GFF files for the WHO and NCTC sequence data are available in the GitHub repository https://github.com/leosanbu/MethylationProject. The *N. gonorrhoeae* FA1090 reference genome was obtained from NCBI accession number AE004969 and the PacBio raw data from the study by Blow *et al*.^6^. Supplementary Table 1 contains the detailed information for each strain.
