# Supplementary material for: Genetic variation regulates the activation and specificity of Restriction-Modification systems in Neisseria gonorrhoeae
Source: Sci Rep. 2019 Oct 11;9:14685. doi: 10.1038/s41598-019-51102-2 (PMC6789123; doi:10.1038/s41598-019-51102-2)
Supplement: Supplementary file 1 — Supplementary Information [file 41598_2019_51102_MOESM1_ESM.pdf]

SUPPLEMENTARY MATERIAL

Genetic variation regulates the activation and specificity of Restriction-Modification systems in *Neisseria gonorrhoeae*

Leonor Sánchez-Busó[1,2,*], Daniel Golparian[3], Julian Parkhill[4], Magnus Unemo[3] and Simon R. Harris[5,*]

[1] Centre for Genomic Pathogen Surveillance, Wellcome Sanger Institute, Wellcome Genome Campus, Hinxton, Cambridge, UK.

[2] Big Data Institute, Nuffield Department of Medicine, University of Oxford, Oxford, UK.

[3] WHO Collaborating Centre for Gonorrhoea and other Sexually Transmitted Infections, National Reference Laboratory for Sexually Transmitted Infections, Department of Laboratory Medicine, Clinical Microbiology, Faculty of Medicine and Health, Örebro University, Örebro, Sweden.

[4] Department of Veterinary Medicine, University of Cambridge, Cambridge, UK.

[5] Microbiotica Ltd, Biodata Innovation Centre, Wellcome Genome Campus, Hinxton, Cambridge, UK.

* To whom correspondence should be addressed. Leonor Sánchez-Busó. Tel: +44 1223 494761; Email: lsb@sanger.ac.uk. Correspondence may also be addressed to Simon R. Harris (sharris@microbiotica.com).

This document contains Supplementary Tables 1-2, 4-9 and Supplementary Figures 1-15.

Supplementary Table 3 is provided as an Excel file.

Supplementary Table 1. List of *N. gonorrhoeae* strains used in the analysis. The ENA accession information of the raw and complete genomic data are indicated along with the average per-base sequencing coverage. Sample accession numbers are used for raw data as some of the strains include more than one sequencing run.

Strain	PacBio data (ENA sample accession)	Genome data accession	Average per-base coverage (X)	References
WHO F	SAMEA2448460	LT591897	118.8	(41)
WHO G	SAMEA2448461	LT591898<br>LT591899<br>LT591900	120.4	(41)
WHO K	SAMEA2448462	LT591908<br>LT591909	173.0	(41)
WHO L	SAMEA2448463	LT591901<br>LT591902<br>LT591903	150.6	(41)
WHO M	SAMEA2448464	LT591904<br>LT591905<br>LT591906<br>LT591907	188.2	(41)
WHO N	SAMEA2448465	LT591910<br>LT591911<br>LT591912<br>LT591913	155.5	(41)
WHO O	SAMEA2448466	LT592146<br>LT592147<br>LT592148<br>LT592149	190.1	(41)
WHO P	SAMEA2448467	LT592157<br>LT592158	211.8	(41)
WHO U	SAMEA2796327	LT592159<br>LT592160	169.9	(41)
WHO V	SAMEA2796328	LT592150<br>LT592151<br>LT592152	142.2	(41)
WHO W	SAMEA2448470	LT592163<br>LT592164<br>LT592165	132.5	(41)
WHO X	SAMEA2448468	LT592155<br>LT592156	204.6	(41)
WHO Y	SAMEA2448469	LT592161<br>LT592162	190.1	(41)
WHO Z	SAMEA2796326	LT592153<br>LT592154	163.7	(41)
NCTC10928	SAMEA3174297	https://github.com/leosanbu/MethylationProject	385.0	NCTC 3000
NCTC10931	SAMEA3174298	https://github.com/leosanbu/MethylationProject	394.0	NCTC 3000
NCTC12700	SAMEA3174299	https://github.com/leosanbu/MethylationProject	425.0	NCTC 3000
NCTC13795	SAMEA4076737	https://github.com/leosanbu/MethylationProject	201.0	NCTC 3000
NCTC13798	SAMEA4076741	https://github.com/leosanbu/MethylationProject	179.0	NCTC 3000
NCTC13799	SAMEA4076765	https://github.com/leosanbu/MethylationProject	149.0	NCTC 3000
NCTC13800	SAMEA4076768	https://github.com/leosanbu/MethylationProject	241.0	NCTC 3000
NCTC13801	SAMEA4076769	https://github.com/leosanbu/MethylationProject	269.0	NCTC 3000
NCTC13802	SAMEA4076770	https://github.com/leosanbu/MethylationProject	185.0	NCTC 3000
NCTC13805	SAMEA4076773	https://github.com/leosanbu/MethylationProject	233.0	NCTC 3000
FA1090	SAMN03775647	NCBI:AE004969	367.0	NCBI, (6)

Supplementary Table 2. Results from a Gene Ontology (GO) enrichment analysis of the flanking genes of the 15 restriction-modifications system (RMS) in *N. gonorrhoeae*. The number of genes annotated in each GO term and the expected number of genes to fall in each category are shown together with the number of significant hits in the flanking genes. GO terms significantly enriched by both the *classic* and *weight01* algorithms (p-value < 0.05) are shown. Results are shown for the three sub-ontologies (BP = Biological Process, MF = Molecular Function, CC = Cellular Component).

**Ontology**	**GO term**	**Term**	**Annotated**	**Significant**	**Expected**	**classic (p-value)**	**weight01 (p-value)**
BP	GO: 0006418	tRNA aminoacylation for protein translation	22	3	0.35	0.0045	0.0045
BP	GO: 0006313	transposition, DNA-mediated	11	2	0.18	0.0123	0.0123
MF	GO: 0004812	aminoacyl-tRNA ligase activity	22	3	0.29	0.0024	0.0024
MF	GO: 0005524	ATP binding	170	6	2.21	0.0167	0.0167
MF	GO: 0000287	magnesium ion binding	20	2	0.26	0.0264	0.0264
MF	GO: 0016853	isomerase activity	57	4	0.74	0.0052	0.0417
CC	GO: 0005737	cytoplasm	165	4	1.39	0.032	0.027
CC	GO: 1902494	catalytic complex	35	2	0.3	0.032	0.032

Supplementary Table 4. Type I NgoAV restriction-modifications system (RMS). Active (A) or disrupted (D) components are indicated. Red highlight is used to mark a cause of disruption. The different sources of variability in the pattern recognition domain of the specificity unit, which cause different methylated motifs (in different colours) are shown. An active methyltransferase requires both the methylase and the specificity unit to be active. In that case, if the restriction enzyme is not functional, the methylase is tagged as 'orphan'. HsdR: Restriction endonuclease; HsdM: Methyltransferase; HsdS: Specificity unit.

	Components								
				Source of variability					
**Strain**	**HsdR**	**HsdM**	**HsdS**	**Premature stop codon**	**Number of LEAT repeats**	**Middle frameshift**	**Orphan HsdM?**	**Active HsdM?**	**Methylated motif**
FA1090	A	A	A	No	2	Yes	No	Yes	GCAN{8}TGC/GCAN{8}TGC
WHO F	D	A	D	No	0	Yes	No	No	-
WHO G	A	A	A	No	2	No	No	Yes	GACN{7}TGC/GCAN{7}GTC
WHO K	A	A	D	Yes	2	Yes	No	No	-
WHO L	A	D	D	Yes	1	Yes	No	No	-
WHO M	A	A	A	No	1	No	No	Yes	GACN{6}TGC/GCAN{6}GTC
WHO N	A	A	A	No	2	No	No	Yes	GACN{7}TGC/GCAN{7}GTC
WHO O	A	A	D	No	0	Yes	No	No	-
WHO P	A	D	D	Yes	3	Yes	No	No	-
WHO U	A	A	A	No	2	No	No	Yes	GACN{7}TGC/GCAN{7}GTC
WHO V	A	A	D	Yes	1	Yes	No	No	-
WHO W	A	A	A	No	2	Yes	No	Yes	GCAN{8}TGC/GCAN{8}TGC
WHO X	A	A	D	Yes	2	Yes	No	No	-
WHO Y	A	A	D	Yes	2	Yes	No	No	-
WHO Z	A	A	D	Yes	2	Yes	No	No	-
NCTC10928	A	A	D	Yes	1	Yes	No	No	-
NCTC10931	A	A	A	No	1	No	No	Yes	GACN{6}TGC/GCAN{6}GTC
NCTC12700	A	A	A	No	1	No	No	Yes	Not detected
NCTC13795	A	D	D	No	1	No	No	No	-
NCTC13798	A	A	D	Yes	2	Yes	No	No	-
NCTC13799	A	D	D	Yes	2	Yes	No	No	-
NCTC13800	A	A	D	Yes	1	Yes	No	No	-
NCTC13801	A	A	A	No	2	No	No	Yes	GACN{7}TGC/GCAN{7}GTC
NCTC13802	A	A	D	Yes	1	Yes	No	No	-
NCTC13805	A	A	D	Yes	1	Yes	No	No	-

Supplementary Table 5. Type I NgoAXVII restriction-modifications system (RMS). Active (A) or disrupted (D, in red) components are indicated. An active methyltransferase requires both the methylase and the specificity unit to be active. In that case, if the restriction enzyme is not functional, the methylase is tagged as 'orphan' (blue). HsdR: Restriction endonuclease; HsdM: Methyltransferase; HsdS: Specificity unit.

	Components					
**Strain**	**HsdR**	**HsdM**	**HsdS**	**Orphan HsdM?**	**Active HsdM?**	**Methylated motif**
FA1090	A	A	A	No	Yes	GAGN{5}TAC/GTAN{5}CTC
WHO F	D	D	A	No	No	-
WHO G	A	A	A	No	Yes	GAGN{5}TAC/GTAN{5}CTC
WHO K	A	A	A	No	Yes	GAGN{5}TAC/GTAN{5}CTC
WHO L	D	A	A	Yes	Yes	GAGN{5}TAC/GTAN{5}CTC
WHO M	A	A	A	No	Yes	GAGN{5}TAC/GTAN{5}CTC
WHO N	A	A	A	No	Yes	GAGN{5}TAC/GTAN{5}CTC
WHO O	A	A	A	No	Yes	GAGN{5}TAC/GTAN{5}CTC
WHO P	A	A	A	No	Yes	GAGN{5}TAC/GTAN{5}CTC
WHO U	A	A	A	No	Yes	GAGN{5}TAC/GTAN{5}CTC
WHO V	A	A	A	No	Yes	GAGN{5}TAC/GTAN{5}CTC
WHO W	A	A	A	No	Yes	GAGN{5}TAC/GTAN{5}CTC
WHO X	A	A	A	No	Yes	GAGN{5}TAC/GTAN{5}CTC
WHO Y	A	A	A	No	Yes	GAGN{5}TAC/GTAN{5}CTC
WHO Z	A	A	A	No	Yes	GAGN{5}TAC/GTAN{5}CTC
NCTC10928	D	A	A	Yes	Yes	GAGN{5}TAC/GTAN{5}CTC
NCTC10931	D	A	A	Yes	Yes	GAGN{5}TAC/GTAN{5}CTC
NCTC12700	A	A	A	No	Yes	GAGN{5}TAC/GTAN{5}CTC
NCTC13795	D	A	A	Yes	Yes	GAGN{5}TAC/GTAN{5}CTC
NCTC13798	A	A	A	No	Yes	GAGN{5}TAC/GTAN{5}CTC
NCTC13799	A	A	A	No	Yes	GAGN{5}TAC/GTAN{5}CTC
NCTC13800	A	A	A	No	Yes	GAGN{5}TAC/GTAN{5}CTC
NCTC13801	D	A	A	Yes	Yes	GAGN{5}TAC/GTAN{5}CTC
NCTC13802	D	A	D	No	No	-
NCTC13805	D	A	D	No	No	-

Supplementary Table 6. Type II restriction-modifications systems (RMS). Active (A) or disrupted (D, in red) components are indicated. An active methyltransferase requires both the methylase and the specificity unit to be active. In that case, if the restriction enzyme is not functional, the methylase is tagged as 'orphan' (blue). R: Restriction enzyme; M: Methyltransferase. The Bonferroni-corrected p-values of a Mann-Whitney test between the IPD values of methylated cytosines of each motif (as indicated in REBASE) and the distribution of unmethylated cytosines for each strain are shown. ****p-value < 0.001; ***p-value<0.001; **p-value<0.01;*p-value<0.05; ns: non-significant.

		Components					Bonferroni-corrected p-values (Cytosine methylation)		
RM	Strain	R	M	Orphan M?	Active M?	Motif	5'-RGCGCY-3'	3'-YCGCGR-5'	(5mC)
NgoAI	FA1090	A	A	No	Yes	RGCGCY	9.47E-93	1.03E-93	****
	WHO F	A	A	No	Yes	RGCGCY	2.17E-96	1.42E-101	****
	WHO G	A	A	No	Yes	RGCGCY	1.75E-80	2.70E-85	****
	WHO K	A	A	No	Yes	RGCGCY	1.94E-95	2.62E-114	****
	WHO L	A	A	No	Yes	RGCGCY	6.70E-86	1.24E-95	****
	WHO M	A	A	No	Yes	RGCGCY	3.40E-110	1.86E-103	****
	WHO N	A	A	No	Yes	RGCGCY	1.07E-97	2.24E-93	****
	WHO O	A	A	No	Yes	RGCGCY	5.19E-114	2.95E-108	****
	WHO P	A	A	No	Yes	RGCGCY	1.88E-107	1.94E-113	****
	WHO U	A	A	No	Yes	RGCGCY	1.05E-87	6.28E-101	****
	WHO V	A	A	No	Yes	RGCGCY	1.44E-98	4.08E-98	****
	WHO W	A	A	No	Yes	RGCGCY	5.43E-93	1.54E-99	****
	WHO X	A	A	No	Yes	RGCGCY	2.01E-103	8.49E-97	****
	WHO Y	A	A	No	Yes	RGCGCY	2.51E-116	5.78E-108	****
	WHO Z	A	A	No	Yes	RGCGCY	1.52E-102	2.98E-97	****
	NCTC10928	A	A	No	Yes	RGCGCY	1.56E-116	7.33E-121	****
	NCTC10931	A	A	No	Yes	RGCGCY	1.56E-123	2.17E-123	****
	NCTC12700	A	A	No	Yes	RGCGCY	5.30E-127	8.16E-114	****
	NCTC13795	A	A	No	Yes	RGCGCY	2.32E-125	1.95E-121	****
	NCTC13798	A	A	No	Yes	RGCGCY	6.24E-130	3.65E-129	****
	NCTC13799	A	A	No	Yes	RGCGCY	1.48E-109	2.78E-121	****
	NCTC13800	A	A	No	Yes	RGCGCY	2.40E-130	2.60E-130	****
	NCTC13801	A	A	No	Yes	RGCGCY	1.81E-131	4.29E-124	****
	NCTC13802	A	A	No	Yes	RGCGCY	1.31E-120	1.83E-136	****
	NCTC13805	A	A	No	Yes	RGCGCY	9.18E-113	3.27E-103	****
RM	Strain	R	M	Orphan M?	Active M?	Motif	5'-GGCC-3'	3'-CCGG-5'	(5mC)
NgoAII	FA1090	D	A	Yes	Yes	GGCC	6.84E-124	1.59E-120	****
	WHO F	D	A	Yes	Yes	GGCC	7.19E-189	3.33E-194	****
	WHO G	A	A	No	Yes	GGCC	3.60E-297	1.42E-305	****
	WHO K	D	A	Yes	Yes	GGCC	5.44E-224	2.08E-238	****
	WHO L	D	A	Yes	Yes	GGCC	3.29E-242	1.42E-248	****
	WHO M	D	A	Yes	Yes	GGCC	1.08E-249	6.61E-286	****
	WHO N	A	A	No	Yes	GGCC	2.12E-235	7.17E-265	****
	WHO O	A	A	No	Yes	GGCC	2.79E-261	3.24E-245	****
	WHO P	D	A	Yes	Yes	GGCC	1.12E-298	0.00E+00	****
	WHO U	D	A	Yes	Yes	GGCC	7.19E-246	6.03E-252	****
	WHO V	D	A	Yes	Yes	GGCC	5.13E-246	8.20E-270	****
	WHO W	D	A	Yes	Yes	GGCC	8.24E-256	4.83E-281	****
	WHO X	D	A	Yes	Yes	GGCC	1.60E-280	6.43E-305	****
	WHO Y	D	A	Yes	Yes	GGCC	0.00E+00	0.00E+00	****
	WHO Z	D	A	Yes	Yes	GGCC	8.33E-229	2.52E-243	****
	NCTC10928	A	A	No	Yes	GGCC	2.17E-73	2.09E-70	****
	NCTC10931	D	A	Yes	Yes	GGCC	8.10E-82	9.35E-75	****
	NCTC12700	A	A	No	Yes	GGCC	5.05E-90	2.10E-76	****
	NCTC13795	A	A	No	Yes	GGCC	3.15E-105	2.62E-80	****
	NCTC13798	D	A	Yes	Yes	GGCC	8.32E-112	7.41E-95	****
	NCTC13799	D	A	Yes	Yes	GGCC	3.70E-125	1.24E-126	****
	NCTC13800	D	A	Yes	Yes	GGCC	1.75E-119	2.18E-114	****
	NCTC13801	A	A	No	Yes	GGCC	3.23E-121	1.68E-102	****
	NCTC13802	D	A	Yes	Yes	GGCC	1.07E-93	4.38E-80	****

RM	Strain	R	M	Orphan M?	Active M?	Motif			
	NCTC13805	D	A	Yes	Yes	GGCC	3.50E-71	5.14E-91	****
**RM**	**Strain**	**R**	**M**	**Orphan M?**	**Active M?**	**Motif**	5'-C**C**GCGG-3'	3'-GGCG**C**C-5'	(5mC)
	FA1090	A	A	No	Yes	CCGCGG	1.30E-23	2.99E-19	****
	WHO F	Absent		No	No	-	1	1	ns
	WHO G	A	A	No	Yes	CCGCGG	6.03E-32	1.36E-33	****
	WHO K	A	A	No	Yes	CCGCGG	3.72E-45	4.85E-36	****
	WHO L	Absent		No	No	-	0.854309627	1	ns
	WHO M	A	A	No	Yes	CCGCGG	5.92E-33	7.52E-34	****
	WHO N	A	A	No	Yes	CCGCGG	8.05E-34	8.89E-25	****
	WHO O	A	A	No	Yes	CCGCGG	7.29E-34	1.23E-25	****
	WHO P	A	A	No	Yes	CCGCGG	9.71E-39	9.15E-36	****
	WHO U	A	A	No	Yes	CCGCGG	8.22E-23	6.74E-26	****
	WHO V	A	A	No	Yes	CCGCGG	2.11E-30	1.97E-29	****
	WHO W	A	A	No	Yes	CCGCGG	3.34E-34	4.23E-33	****
NgoAIII	WHO X	A	A	No	Yes	CCGCGG	4.70E-43	2.94E-38	****
	WHO Y	A	A	No	Yes	CCGCGG	2.84E-43	1.21E-37	****
	WHO Z	A	A	No	Yes	CCGCGG	5.06E-28	7.33E-19	****
	NCTC10928	Absent		No	No	-	1	0.125551747	ns
	NCTC10931	A	A	No	Yes	CCGCGG	2.20E-01	0.031189686	ns/*
	NCTC12700	Absent		No	No	-	2.29E-06	0.000732929	****/***
	NCTC13795	A	A	No	Yes	CCGCGG	2.21E-02	0.001653781	*/**
	NCTC13798	A	A	No	Yes	CCGCGG	1.81E-03	0.023353729	**/*
	NCTC13799	A	A	No	Yes	CCGCGG	1.12E-02	0.003917145	*/**
	NCTC13800	A	A	No	Yes	CCGCGG	1.05E-03	0.000816589	**/***
	NCTC13801	A	A	No	Yes	CCGCGG	5.84E-04	0.000900113	***
	NCTC13802	A	A	No	Yes	CCGCGG	4.41E-04	0.002522283	***/**
	NCTC13805	A	A	No	Yes	CCGCGG	1.69E-01	0.024751118	ns/*
**RM**	**Strain**	**R**	**M**	**Orphan M?**	**Active M?**	**Motif**	5'-G**C**CGGC-3'	3'-CGGC**C**G-5'	(5mC)
	FA1090	A	A	No	Yes	GCCGGC	1.77E-271	4.52E-263	****
	WHO F	A	A	No	Yes	GCCGGC	3.53E-203	3.75E-218	****
	WHO G	A	A	No	Yes	GCCGGC	1.26E-289	1.79E-272	****
	WHO K	A	A	No	Yes	GCCGGC	2.23E-253	1.30E-229	****
	WHO L	A	A	No	Yes	GCCGGC	5.89E-226	2.81E-270	****
	WHO M	A	A	No	Yes	GCCGGC	7.95E-274	3.23E-271	****
	WHO N	A	A	No	Yes	GCCGGC	1.19E-261	8.17E-253	****
	WHO O	A	A	No	Yes	GCCGGC	2.99E-280	2.44E-265	****
	WHO P	A	A	No	Yes	GCCGGC	0.00E+00	0.00E+00	****
	WHO U	A	A	No	Yes	GCCGGC	0.00E+00	0.00E+00	****
	WHO V	A	A	No	Yes	GCCGGC	1.67E-268	2.02E-268	****
	WHO W	A	A	No	Yes	GCCGGC	2.24E-276	4.76E-246	****
NgoAIV	WHO X	A	A	No	Yes	GCCGGC	2.00E-290	1.13E-284	****
	WHO Y	A	A	No	Yes	GCCGGC	0.00E+00	0.00E+00	****
	WHO Z	A	A	No	Yes	GCCGGC	0.00E+00	0.00E+00	****
	NCTC10928	A	A	No	Yes	GCCGGC	0.00E+00	0.00E+00	****
	NCTC10931	A	A	No	Yes	GCCGGC	0.00E+00	0.00E+00	****
	NCTC12700	A	A	No	Yes	GCCGGC	0.00E+00	0.00E+00	****
	NCTC13795	A	A	No	Yes	GCCGGC	0.00E+00	0.00E+00	****
	NCTC13798	A	A	No	Yes	GCCGGC	0.00E+00	0.00E+00	****
	NCTC13799	A	A	No	Yes	GCCGGC	0.00E+00	0.00E+00	****
	NCTC13800	A	A	No	Yes	GCCGGC	0.00E+00	0.00E+00	****
	NCTC13801	A	A	No	Yes	GCCGGC	0.00E+00	0.00E+00	****
	NCTC13802	A	A	No	Yes	GCCGGC	0.00E+00	0.00E+00	****
	NCTC13805	A	A	No	Yes	GCCGGC	0.00E+00	0.00E+00	****

RM	Strain	R	M	Orphan M?	Active M?	Motif	5'-GCGGC-3'	3'-CGCCG-5'	(5mC)
NgoAVII	FA1090	A	A	No	Yes	GCGGC	7.56E-123	6.17E-68	****
	WHO F	A	A	No	Yes	GCGGC	3.78E-92	3.32E-22	****
	WHO G	A	A	No	Yes	GCGGC	1.00E+00	3.72E-58	ns/****
	WHO K	A	A	No	Yes	GCGGC	7.09E-119	5.88E-48	****
	WHO L	A	A	No	Yes	GCGGC	1.06E-54	9.90E-63	****
	WHO M	A	A	No	Yes	GCGGC	5.88E-54	2.33E-48	****
	WHO N	A	A	No	Yes	GCGGC	2.46E-49	2.83E-51	****
	WHO O	A	A	No	Yes	GCGGC	6.76E-120	1.67E-65	****
	WHO P	A	A	No	Yes	GCGGC	7.85E-102	2.67E-74	****
	WHO U	A	A	No	Yes	GCGGC	4.22E-19	6.56E-108	****
	WHO V	A	A	No	Yes	GCGGC	1.87E-85	5.11E-103	****
	WHO W	A	A	No	Yes	GCGGC	8.73E-148	3.31E-89	****
	WHO X	A	A	No	Yes	GCGGC	1.90E-95	6.45E-42	****
	WHO Y	A	A	No	Yes	GCGGC	0.00E+00	0.00E+00	****
	WHO Z	A	A	No	Yes	GCGGC	0.00E+00	0.00E+00	****
	NCTC10928	A	A	No	Yes	GCGGC	2.40E-234	0.00E+00	****
	NCTC10931	A	A	No	Yes	GCGGC	5.16E-237	0.00E+00	****
	NCTC12700	A	A	No	Yes	GCGGC	0.00E+00	0.00E+00	****
	NCTC13795	A	A	No	Yes	GCGGC	2.07E-289	0.00E+00	****
	NCTC13798	A	A	No	Yes	GCGGC	0.00E+00	0.00E+00	****
	NCTC13799	A	A	No	Yes	GCGGC	0.00E+00	0.00E+00	****
	NCTC13800	A	A	No	Yes	GCGGC	0.00E+00	0.00E+00	****
	NCTC13801	A	A	No	Yes	GCGGC	0.00E+00	0.00E+00	****
	NCTC13802	A	A	No	Yes	GCGGC	0.00E+00	0.00E+00	****
	NCTC13805	A	A	No	Yes	GCGGC	0.00E+00	0.00E+00	****

RM	Strain	RM	S	Orphan M?	Active M?	Motif
NgoAVIII	FA1090	A	A	No	Yes	Unknown
	WHO F	A	A	No	Yes	Unknown
	WHO G	A	A	No	Yes	Unknown
	WHO K	A	A	No	Yes	Unknown
	WHO L	A	A	No	Yes	Unknown
	WHO M	A	A	No	Yes	Unknown
	WHO N	A	A	No	Yes	Unknown
	WHO O	A	A	No	Yes	Unknown
	WHO P	A	A	No	Yes	Unknown
	WHO U	A	A	No	Yes	Unknown
	WHO V	A	A	No	Yes	Unknown
	WHO W	A	A	No	Yes	Unknown
	WHO X	A	A	No	Yes	Unknown
	WHO Y	A	A	No	Yes	Unknown
	WHO Z	A	A	No	Yes	Unknown
	NCTC10928	A	A	No	Yes	Unknown
	NCTC10931	A	A	No	Yes	Unknown
	NCTC12700	A	A	No	Yes	Unknown
	NCTC13795	A	A	No	Yes	Unknown
	NCTC13798	A	A	No	Yes	Unknown
	NCTC13799	A	A	No	Yes	Unknown
	NCTC13800	A	A	No	Yes	Unknown
	NCTC13801	A	A	No	Yes	Unknown
	NCTC13802	A	A	No	Yes	Unknown
	NCTC13805	A	A	No	Yes	Unknown

RM	Strain	VSR	M	Orphan M?	Active M?	Motif
NgoAXIII	FA1090	-	D	No	No	-
	WHO F	A	D	No	No	-
	WHO G	A	D	No	No	-
	WHO K	A	D	No	No	-
	WHO L	D	D	No	No	-
	WHO M	A	D	No	No	-
	WHO N	A	D	No	No	-
	WHO O	A	D	No	No	-
	WHO P	A	D	No	No	-
	WHO U	A	D	No	No	-
	WHO V	A	D	No	No	-
	WHO W	A	D	No	No	-
	WHO X	A	D	No	No	-
	WHO Y	A	D	No	No	-
	WHO Z	A	D	No	No	-
	NCTC10928	A	D	No	No	-
	NCTC10931	A	D	No	No	-
	NCTC12700	A	D	No	No	-
	NCTC13795	A	D	No	No	-
	NCTC13798	A	D	No	No	-
	NCTC13799	A	D	No	No	-
	NCTC13800	A	D	No	No	-
	NCTC13801	A	D	No	No	-
	NCTC13802	A	D	No	No	-
	NCTC13805	A	D	No	No	-

RM	Strain	R	M	Orphan M?	Active M?	Motif
NgoAXI	FA1090	A	D	No	No	-
	WHO F	A	D	No	No	-
	WHO G	A	D	No	No	-
	WHO K	A	D	No	No	-
	WHO L	A	D	No	No	-
	WHO M	A	D	No	No	-
	WHO N	A	D	No	No	-
	WHO O	A	D	No	No	-
	WHO P	A	D	No	No	-
	WHO U	A	D	No	No	-
	WHO V	A	D	No	No	-
	WHO W	A	D	No	No	-
	WHO X	A	D	No	No	-
	WHO Y	A	D	No	No	-
	WHO Z	A	D	No	No	-
	NCTC10928	A	D	No	No	-
	NCTC10931	Dam	D	Yes	Yes	GATC
	NCTC12700	Dam	D	Yes	Yes	GATC
	NCTC13795	A	D	No	No	-
	NCTC13798	A	D	No	No	-
	NCTC13799	A	D	No	No	-
	NCTC13800	A	D	No	No	-
	NCTC13801	A	D	No	No	-
	NCTC13802	A	D	No	No	-
	NCTC13805	A	D	No	No	-

RM	Strain	VSR	M	Orphan M?	Active M?	Motif	5'-CCGG-3'	3-GGCC-5'	
	FA1090	A	A	No	Yes	CCGG	1.95E-41	6.38E-47	****
	WHO F	A	A	No	Yes	CCGG	1.47E-120	1.71E-124	****
	WHO G	A	A	No	Yes	CCGG	5.05E-29	2.68E-28	****
	WHO K	A	A	No	Yes	CCGG	3.37E-119	1.03E-117	****
	WHO L	A	A	No	Yes	CCGG	8.86E-56	1.53E-94	****
	WHO M	A	A	No	Yes	CCGG	7.51E-79	1.15E-92	****
	WHO N	A	A	No	Yes	CCGG	2.21E-51	4.35E-59	****
	WHO O	A	A	No	Yes	CCGG	4.09E-123	1.30E-119	****
	WHO P	A	A	No	Yes	CCGG	3.27E-107	2.54E-111	****
	WHO U	A	A	No	Yes	CCGG	0.040205158	0.005377179	*/**
	WHO V	A	A	No	Yes	CCGG	1.63E-19	4.60E-21	****
	WHO W	A	A	No	Yes	CCGG	1.52E-64	3.32E-82	****
NgoAXIV	WHO X	A	A	No	Yes	CCGG	3.61E-143	7.17E-151	****
	WHO Y	A	A	No	Yes	CCGG	3.30E-129	1.47E-144	****
	WHO Z	A	A	No	Yes	CCGG	3.78E-07	9.17E-14	****
	NCTC10928	A	A	No	Yes	CCGG	2.02E-36	2.12E-47	****
	NCTC10931	A	A	No	Yes	CCGG	1.38E-27	9.10E-45	****
	NCTC12700	A	A	No	Yes	CCGG	7.13E-44	3.96E-50	****
	NCTC13795	A	A	No	Yes	CCGG	1.05E-62	7.93E-75	****
	NCTC13798	A	A	No	Yes	CCGG	9.85E-54	1.69E-78	****
	NCTC13799	A	A	No	Yes	CCGG	2.30E-105	1.38E-123	****
	NCTC13800	A	A	No	Yes	CCGG	8.55E-73	6.82E-105	****
	NCTC13801	A	A	No	Yes	CCGG	5.43E-72	8.88E-79	****
	NCTC13802	A	A	No	Yes	CCGG	2.99E-89	1.35E-63	****
	NCTC13805	A	A	No	Yes	CCGG	8.87E-55	1.01E-81	****

RM	Strain	R	M	Orphan M?	Active M?	Motif	5'-GGNNCC-3'	3-CCNNGG-5'	(5mC)
	FA1090	A	A	No	Yes	GGNNCC	8.84E-252	1.46E-234	****
	WHO F	A	A	No	Yes	GGNNCC	1.05E-200	5.33E-191	****
	WHO G	D	A	Yes	Yes	GGNNCC	0.00E+00	0.00E+00	****
	WHO K	A	A	No	Yes	GGNNCC	1.23E-226	6.68E-209	****
	WHO L	A	A	No	Yes	GGNNCC	2.33E-207	3.27E-242	****
	WHO M	D	A	Yes	Yes	GGNNCC	4.70E-264	2.45E-252	****
	WHO N	D	A	Yes	Yes	GGNNCC	6.64E-223	1.54E-245	****
	WHO O	D	A	Yes	Yes	GGNNCC	1.42E-247	4.20E-237	****
	WHO P	D	A	Yes	Yes	GGNNCC	7.06E-291	1.11E-284	****
	WHO U	D	A	Yes	Yes	GGNNCC	2.60E-283	6.29E-280	****
	WHO V	A	A	No	Yes	GGNNCC	1.15E-259	8.75E-266	****
	WHO W	A	A	No	Yes	GGNNCC	2.18E-234	2.95E-231	****
NgoAXV	WHO X	A	A	No	Yes	GGNNCC	1.14E-240	2.58E-258	****
	WHO Y	A	A	No	Yes	GGNNCC	1.51E-279	1.35E-297	****
	WHO Z	A	A	No	Yes	GGNNCC	1.78E-245	3.09E-267	****
	NCTC10928	A	A	No	Yes	GGNNCC	7.72E-134	2.68E-158	****
	NCTC10931	A	A	No	Yes	GGNNCC	1.64E-152	4.17E-168	****
	NCTC12700	A	A	No	Yes	GGNNCC	1.27E-153	1.73E-166	****
	NCTC13795	A	A	No	Yes	GGNNCC	3.19E-154	1.77E-159	****
	NCTC13798	A	A	No	Yes	GGNNCC	6.07E-164	2.31E-168	****
	NCTC13799	D	A	Yes	Yes	GGNNCC	4.22E-170	5.46E-186	****
	NCTC13800	A	A	No	Yes	GGNNCC	4.09E-179	3.20E-175	****
	NCTC13801	A	A	No	Yes	GGNNCC	1.29E-168	2.33E-170	****
	NCTC13802	A	A	No	Yes	GGNNCC	1.45E-157	7.76E-148	****
	NCTC13805	A	A	No	Yes	GGNNCC	6.37E-135	3.72E-160	****

RM	Strain	R	M	Orphan M?	Active M?	Motif
NgoAXVI	FA1090	D	A	Yes	Yes	GGTGA
	WHO F	D	A	Yes	Yes	GGTGA
	WHO G	A	A	No	Yes	GGTGA
	WHO K	A	A	No	Yes	GGTGA
	WHO L	A	A	No	Yes	GGTGA
	WHO M	A	A	No	Yes	GGTGA
	WHO N	A	A	No	Yes	GGTGA
	WHO O	D	A	Yes	Yes	GGTGA
	WHO P	A	A	No	Yes	GGTGA
	WHO U	A	A	No	Yes	GGTGA
	WHO V	A	A	No	Yes	GGTGA
	WHO W	A	A	No	Yes	GGTGA
	WHO X	A	A	No	Yes	GGTGA
	WHO Y	A	A	No	Yes	GGTGA
	WHO Z	A	A	No	Yes	GGTGA
	NCTC10928	A	A	No	Yes	GGTGA
	NCTC10931	A	A	No	Yes	GGTGA
	NCTC12700	A	A	No	Yes	GGTGA
	NCTC13795	A	A	No	Yes	GGTGA
	NCTC13798	A	A	No	Yes	GGTGA
	NCTC13799	A	A	No	Yes	GGTGA
	NCTC13800	A	A	No	Yes	GGTGA
	NCTC13801	A	A	No	Yes	GGTGA
	NCTC13802	A	A	No	Yes	GGTGA
	NCTC13805	A	A	No	Yes	GGTGA

Supplementary Table 7. Type III NgoAX restriction-modifications system (RMS). Active (A) or disrupted (D, in red) components are indicated. The number of CCCAA repeats and the DNA recognition domain (DRD) allele are shown, which are the source of variability in the Mod unit. An active methyltransferase requires both the methylase and the specificity unit to be active. In that case, if the restriction enzyme is not functional, the methylase is tagged as 'orphan'. Res = Restriction enzyme, Mod = Methyltransferase.

			Source of variability				
**Strain**	**Res**	**Mod**	**Number of CCCAA repeats**	**DRD allele**	**Orphan Mod?**	**Active Mod?**	**Motif**
FA1090	A	A	12	*modB1*	No	Yes	CCACC
WHO F	A	A	6	*modB1*	No	Yes	CCACC
WHO G	A	D	13	*modB1*	No	No	-
WHO K	A	D	10	*modB1*	No	No	-
WHO L	A	D	5	*modB1*	No	No	-
WHO M	A	D	8	*modB1*	No	No	-
WHO N	A	D	13	*modB1*	No	No	-
WHO O	A	D	7	*modB1*	No	No	-
WHO P	A	A	18	*modB1*	No	Yes	CCACC
WHO U	A	D	8	*modB1*	No	No	-
WHO V	A	D	10	*modB1*	No	No	-
WHO W	A	D	8	*modB1*	No	No	-
WHO X	A	D	8	*modB1*	No	No	-
WHO Y	A	A	9	*modB1*	No	Yes	CCACC
WHO Z	A	D	5	*modB1*	No	No	-
NCTC10928	A	D	19	*modB1*	No	No	-
NCTC10931	A	D	7	*modB1*	No	No	-
NCTC12700	A	A	36	*modB1*	No	Yes	CCACC
NCTC13795	A	D	7	*modB1*	No	No	-
NCTC13798	A	A	9	*modB1*	No	Yes	CCACC
NCTC13799	A	D	4	*modB1*	No	No	-
NCTC13800	A	D	7	*modB1*	No	No	-
NCTC13801	A	D	10	*modB1*	No	No	-
NCTC13802	A	D	7	*modB1*	No	No	-
NCTC13805	A	D	7	*modB1*	No	No	-

Supplementary Table 8. Type III NgoAXII restriction-modifications system (RMS). Active (A) or disrupted (D, in red) components are indicated. The number of AGCC repeats and the DNA-recognition domain (DRD) allele are shown, which are the source of variability in the Mod unit. An active methyltransferase requires both the methylase and the specificity unit to be active. In that case, if the restriction enzyme is not functional, the methylase is tagged as 'orphan'. Res = Restriction enzyme, Mod = Methyltransferase.

Strain	Res	Mod	Source of variability		Orphan Mod?	Active Mod?	Motif
			Number of AGCC repeats	DRD allele			
FA1090	A	D	35	*modA13*	No	No	-
WHO F	A	D	22	*modA13*	No	No	-
WHO G	A	D	14	*modA13*	No	No	-
WHO K	A	D	23	*modA13*	No	No	-
WHO L	A	D	14	*modA13*	No	No	-
WHO M	A	D	12	*modA13*	No	No	-
WHO N	A	A	28	*modA13*	No	Yes	GCAGA
WHO O	A	D	15	*modA13*	No	No	-
WHO P	A	D	15	*modA13*	No	No	-
WHO U	A	D	15	*modA13*	No	No	-
WHO V	A	D	18	*modA13*	No	No	-
WHO W	A	D	20	*modA13*	No	No	-
WHO X	A	D	21	*modA13*	No	No	-
WHO Y	A	D	18	*modA13*	No	No	-
WHO Z	A	D	18	*modA13*	No	No	-
NCTC10928	A	D	14	*modA13*	No	No	-
NCTC10931	A	D	18	*modA13*	No	No	-
NCTC12700	A	D	15	*modA13*	No	No	-
NCTC13795	A	D	25	*modA13*	No	No	-
NCTC13798	A	A	13	*modA13*	No	Yes	GCAGA
NCTC13799	A	A	19	*modA13*	No	Yes	GCAGA
NCTC13800	A	D	17	*modA13*	No	No	-
NCTC13801	A	D	12	*modA13*	No	No	-
NCTC13802	A	D	3	*modA13*	No	No	-
NCTC13805	A	D	3	*modA13*	No	No	-

Supplementary Table 9. Number of GCAGA and AGAAA and GCAGAAATTY motifs in each strain and number and proportion of those with an IPD>3 in the following underlined bases. GCAGA is the motif associated to NgoAXII detected by the PacBio SMRT analysis pipeline (NCTC13798, NCTC13799 and WHO N), AGAAA is the predicted motif for this methylase in REBASE and GCAGAAATTY is the motif resulting from overlapping GCAGA, AGAAA and the recognition sequence of the ApoI enzyme (5'-RAATTY-3').

	GCAGA			AGAAA			GCAGAAATTY				
	Number of motifs	Number of motifs with IPD>3 in GCAGA	% methylated motifs (IPD>3)	Number of motifs	Number of motifs with IPD>3 in AGAAA	% methylated motifs (IPD>3)	Number of motifs	Number of motifs with IPD>3 in GCAGAAATTY	% methylated GCAGAAATTY (IPD>3)	Number of motifs with IPD>3 in GCAGAAATTY	% methylated GCAGAAATTY (IPD>3)
FA1090	4560	3	0.07	5134	0	0.00	5	0	0.00	0	0.00
NCTC10928	4779	9	0.19	5449	1	0.02	5	0	0.00	0	0.00
NCTC10931	4984	111	2.23	5938	0	0.00	7	0	0.00	0	0.00
NCTC12700	4628	77	1.66	5250	0	0.00	5	0	0.00	0	0.00
NCTC13795	4672	12	0.26	5337	2	0.04	6	0	0.00	0	0.00
NCTC13798	4746	3970	83.65	5507	1	0.02	6	4	66.67	0	0.00
NCTC13799	4611	4169	90.41	5288	2	0.04	5	3	60.00	0	0.00
NCTC13800	4740	10	0.21	5498	0	0.00	5	0	0.00	0	0.00
NCTC13801	4739	32	0.68	5483	0	0.00	5	0	0.00	0	0.00
NCTC13802	4842	10	0.21	5568	0	0.00	8	0	0.00	0	0.00
NCTC13805	5110	11	0.22	5938	1	0.02	7	0	0.00	0	0.00
WHO F	4856	2	0.04	5728	0	0.00	8	0	0.00	0	0.00
WHO G	4742	29	0.61	5447	0	0.00	4	0	0.00	0	0.00
WHO K	4615	8	0.17	5254	0	0.00	5	0	0.00	0	0.00
WHO L	4737	9	0.19	5360	0	0.00	5	0	0.00	0	0.00
WHO M	4783	35	0.73	5480	0	0.00	4	0	0.00	0	0.00
WHO N	4772	3791	79.44	5499	0	0.00	4	3	75.00	0	0.00
WHO O	4758	7	0.15	5432	0	0.00	5	0	0.00	0	0.00
WHO P	4630	5	0.11	5277	0	0.00	5	0	0.00	0	0.00
WHO U	4760	26	0.55	5551	1	0.02	5	0	0.00	0	0.00
WHO V	4754	2	0.04	5517	0	0.00	4	0	0.00	0	0.00
WHO W	4893	7	0.14	5613	0	0.00	5	0	0.00	0	0.00
WHO X	4624	6	0.13	5312	0	0.00	4	0	0.00	0	0.00
WHO Y	4765	8	0.17	5510	0	0.00	5	0	0.00	0	0.00
WHO Z	4773	12	0.25	5498	0	0.00	4	0	0.00	0	0.00

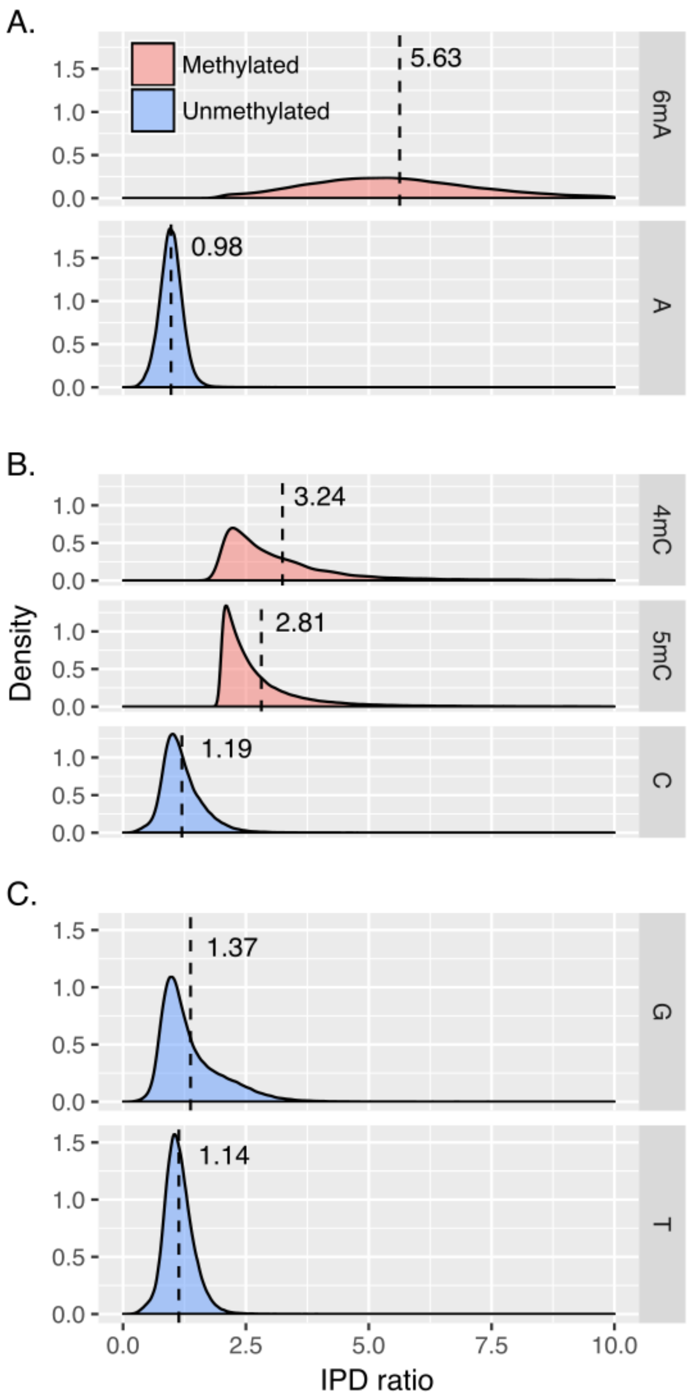

Supplementary Figure 1. Distribution of the IPD ratios for methylated and unmethylated bases. 6mA, 4mC and 5mC IPD values were extracted from the target bases in the final list of curated motifs. Those below an IPD ratio of 2 where excluded to minimize the inclusion of unmethylated motifs. Values for each unmethylated base correspond to a random sample of 10,000 sites per strain outside all the non-redundant predicted motifs detected by the PacBio SMRT pipeline. Dashed vertical lines and numbers indicate the mean value of each distribution.

.

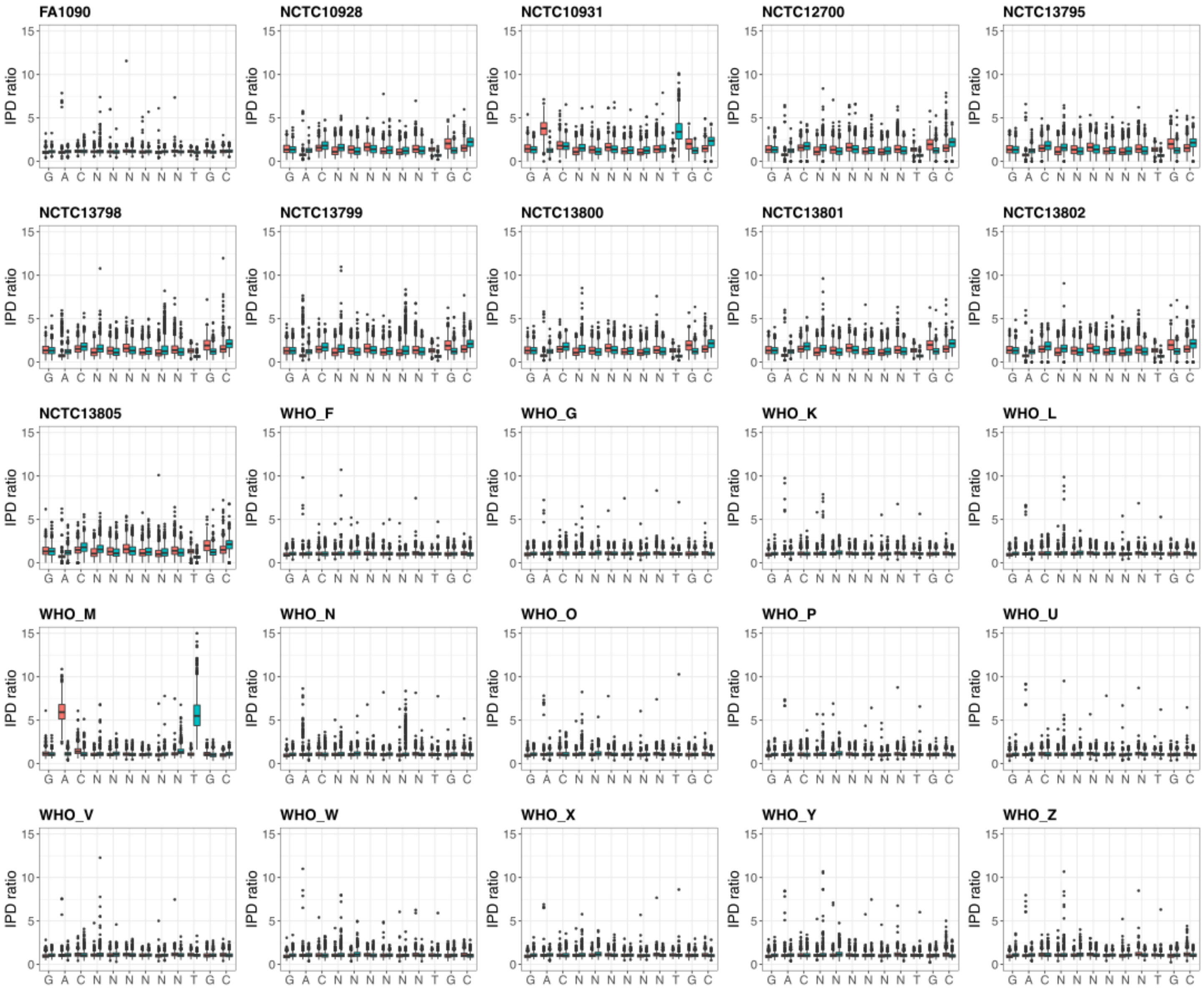

Supplementary Figure 2. IPD ratio values for each base in all instances of the 5’-GACN{6}TGC-3’ motif, target of the Type I NgoAV RMS, in each of the 25 *N. gonorrhoeae* strains included in the study. Per-base distribution of IPD ratio is shown as two separate boxplots containing the values in the forward (red) and reverse (blue) strand.

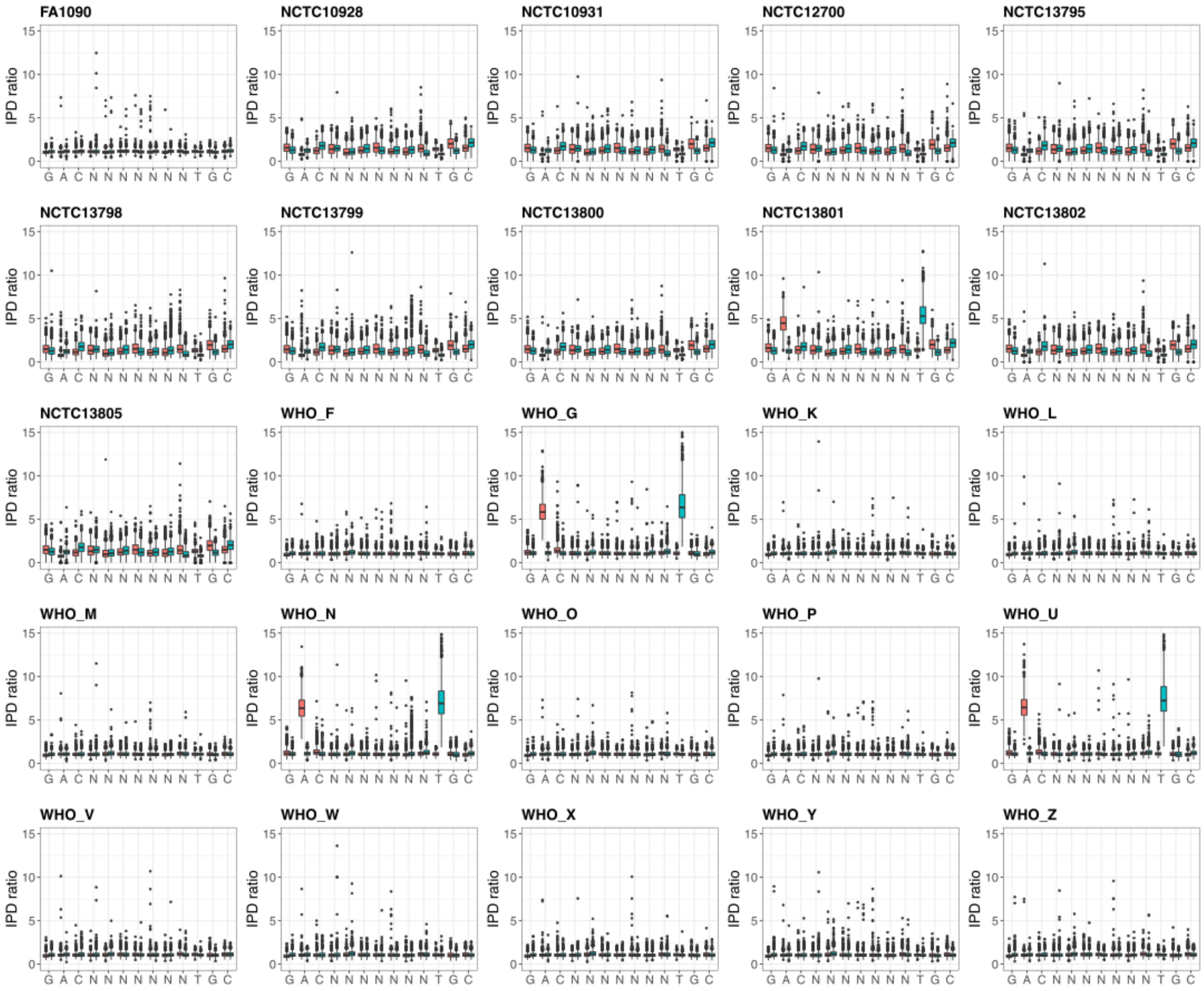

Supplementary Figure 3. IPD ratio values for each base in all instances of the 5'-GACN{7}TGC-3' motif, target of the Type I NgoAV RMS, in each of the 25 *N. gonorrhoeae* strains included in the study. Per-base distribution of IPD ratio is shown as two separate boxplots containing the values in the forward (red) and reverse (blue) strand.

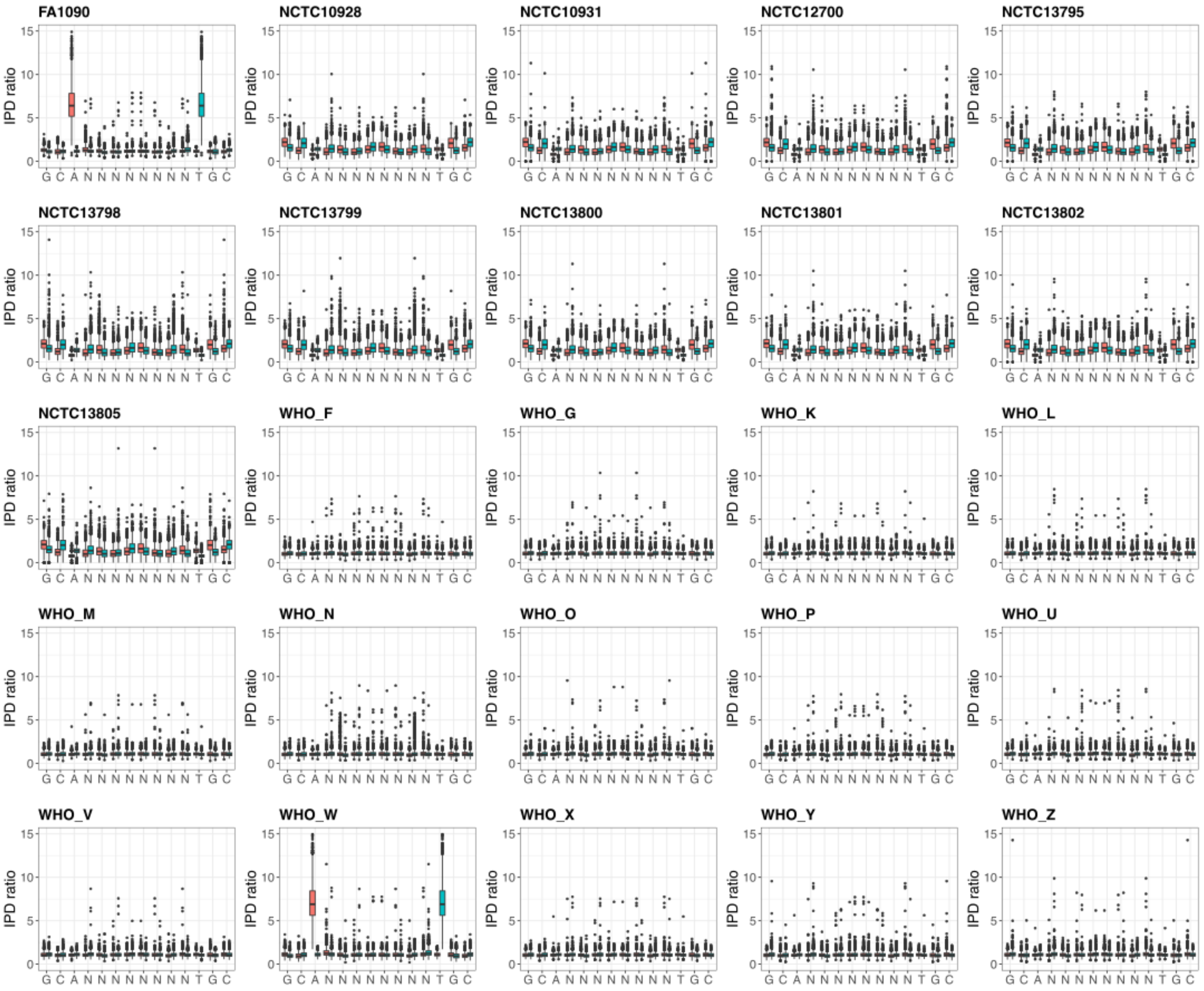

Supplementary Figure 4. IPD ratio values for each base in all instances of the 5’-GCAN{8}TGC-3’ motif, target of the Type I NgoAV RMS, in each of the 25 *N. gonorrhoeae* strains included in the study. Per-base distribution of IPD ratio is shown as two separate boxplots containing the values in the forward (red) and reverse (blue) strand.

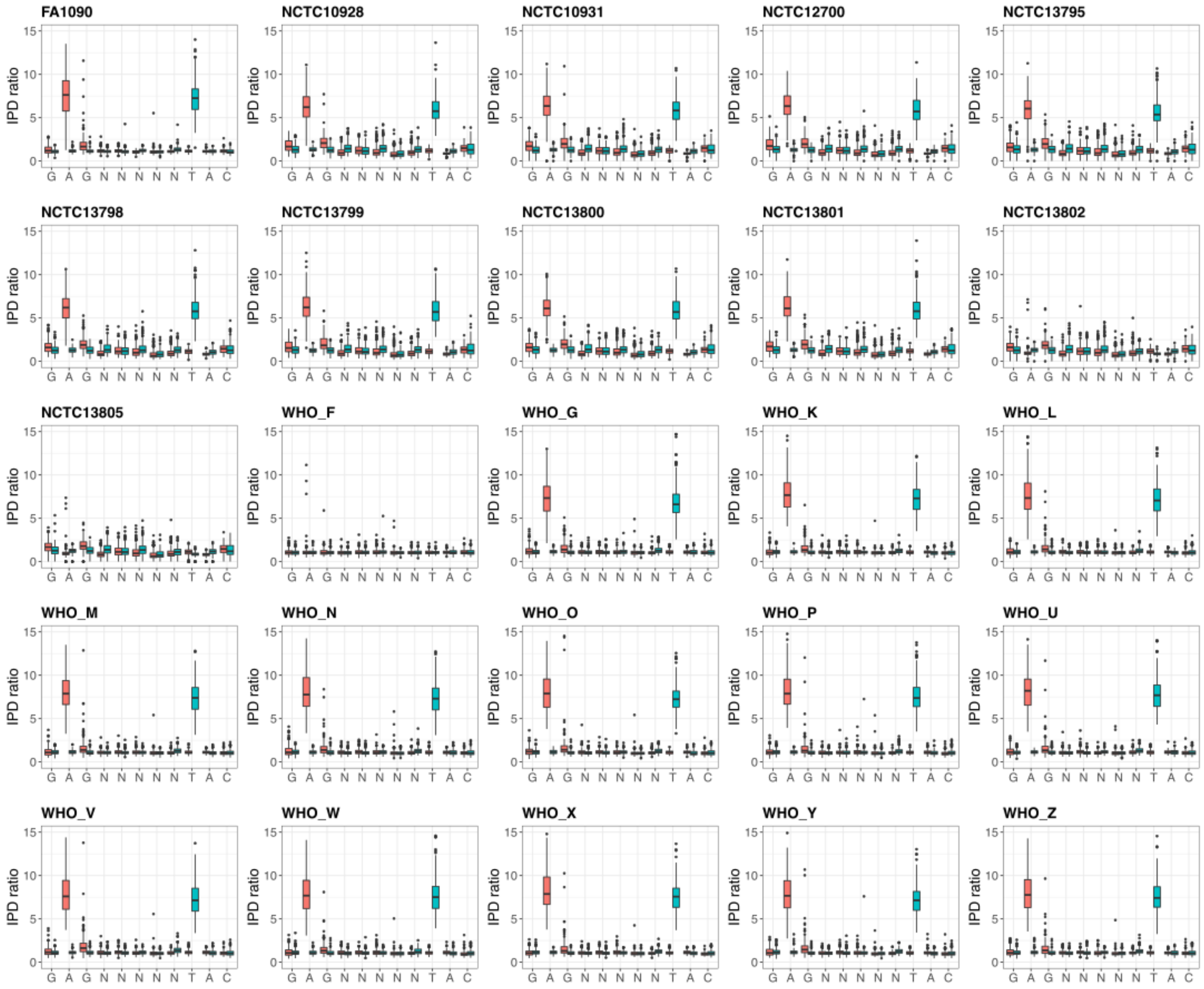

Supplementary Figure 5. IPD ratio values for each base in all instances of the 5'-GAGN{5}TAC-3' motif, target of the Type I NgoAXVII RMS, in each of the 25 *N. gonorrhoeae* strains included in the study. Per-base distribution of IPD ratio is shown as two separate boxplots containing the values in the forward (red) and reverse (blue) strand.

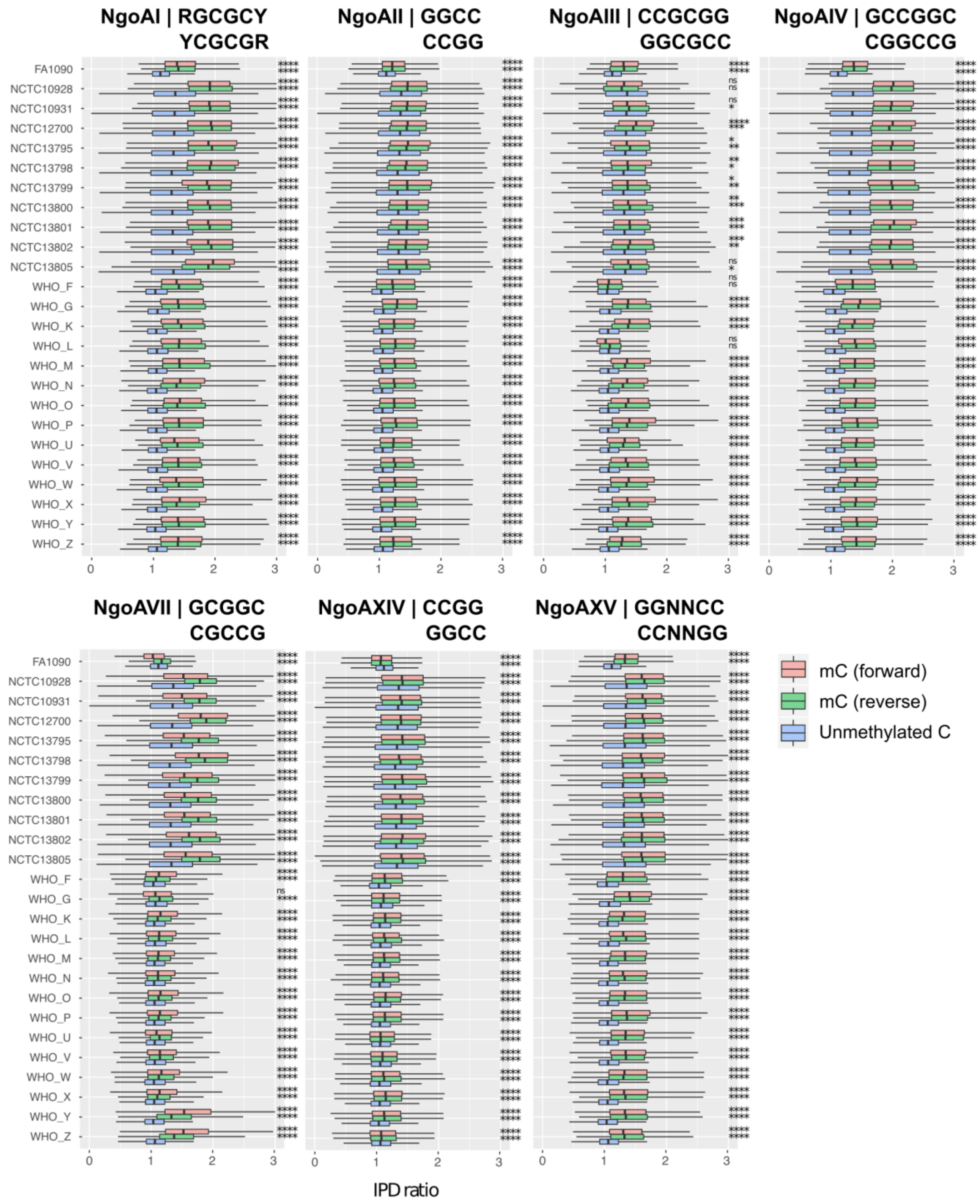

Supplementary Figure 6. IPD ratios for methylated cytosines (mCs) in Type II restriction-modifications system (RMS). The distribution of IPD ratios for the forward (red) and reverse (green) mCs as stated in REBASE are shown as boxplots together with a random 10k subsampling of unmethylated cytosines for each strain (blue). Statistical significance of the comparison among the distribution of the forward and reverse methylations with that from a random 10k subsampling of unmethylated cytosines is indicated next to the boxplots. ****p-value < 0.0001; ***p-value<0.001; **p-value<0.01;*p-value<0.05; ns: non-significant.

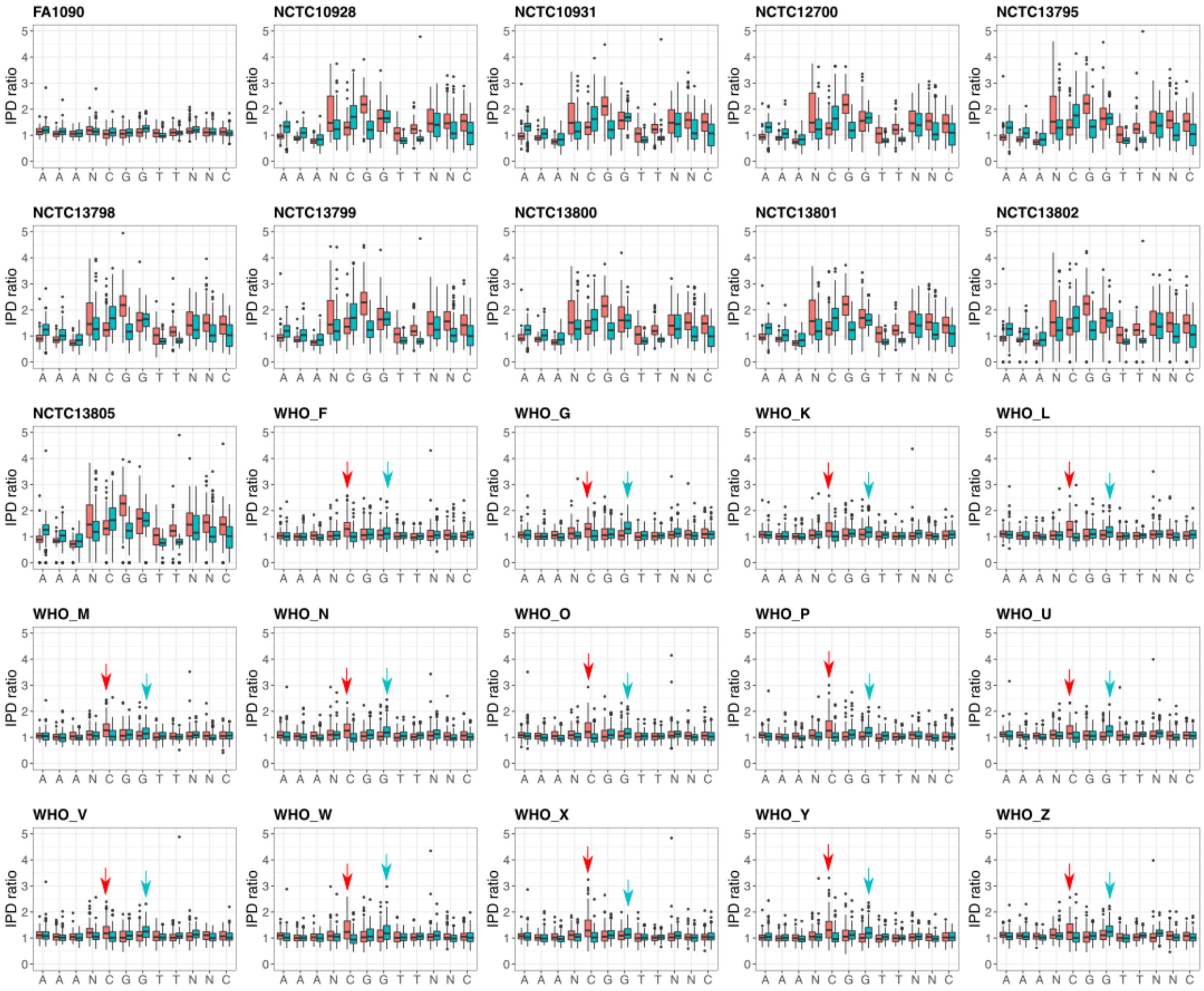

Supplementary Figure 7. IPD ratio values for each base in all instances of the 5'-AAANCGGTTNNC-3' motif in each of the 25 *N. gonorrhoeae* strains included in the study. Per-base distribution of IPD ratio is shown as two separate boxplots containing the values in the forward (red) and reverse (blue) strand.

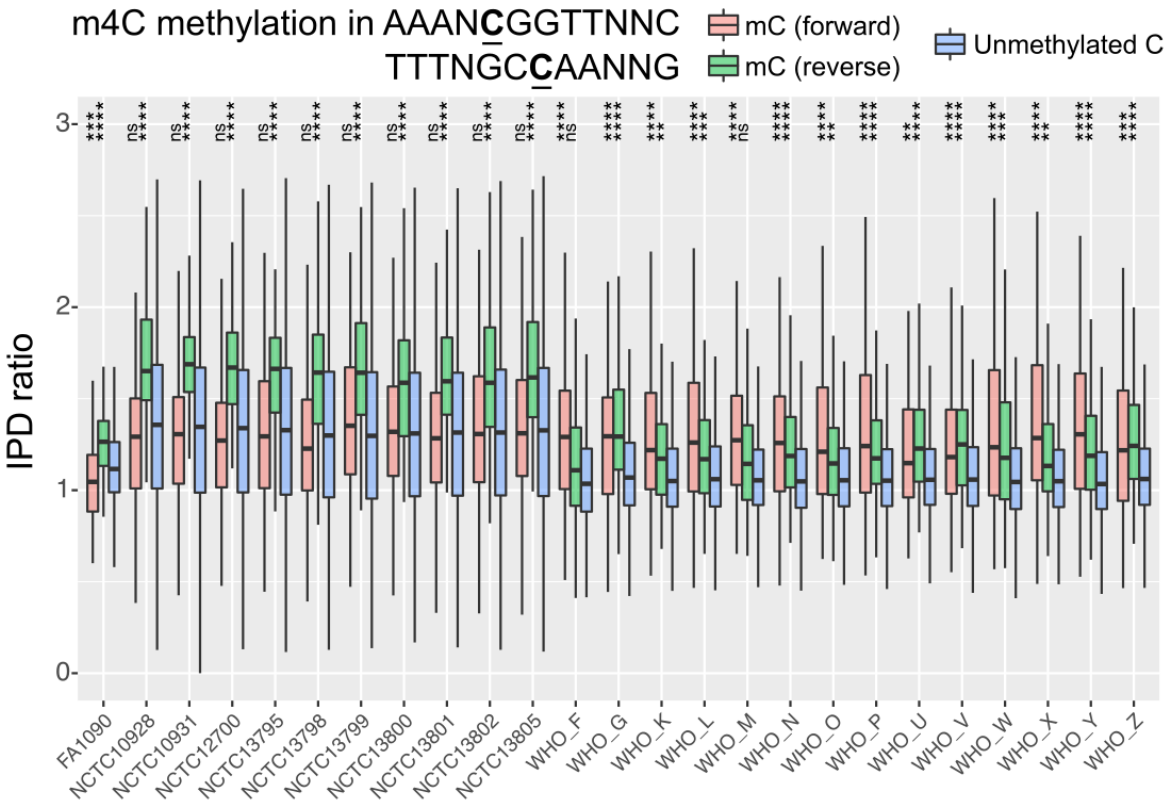

Supplementary Figure 8. IPD ratios for the underlined forward and reverse cytosines in the 5'-AAANCGGTTNNC-3' motif. These values were compared to the distribution of a random 10k subsampling of unmethylated cytosines for each strain. Statistical significance is shown above the boxplots.

****p-value < 0.0001; ***p-value<0.001; **p-value<0.01;*p-value<0.05; ns: non-significant.

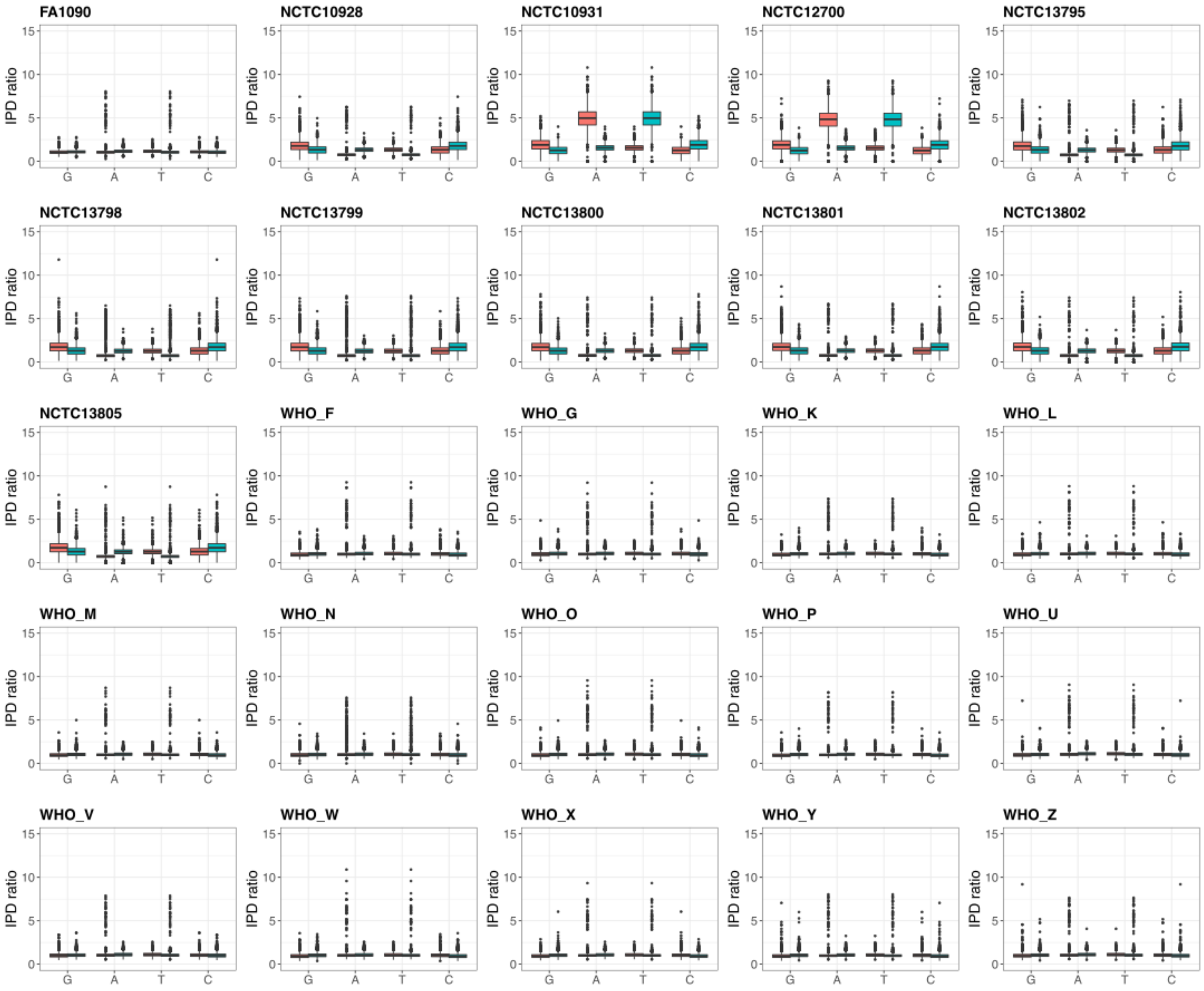

Supplementary Figure 9. IPD ratio values for each base in all instances of the 5'-GATC-3' motif, target of the Type II NgoAXI RMS, in each of the 25 *N. gonorrhoeae* strains included in the study. Per-base distribution of IPD ratio is shown as two separate boxplots containing the values in the forward (red) and reverse (blue) strand.

Supplementary Figure 10. IPD ratio values for each base in all instances of the 5’-GGTGA-3’ motif, target of the Type II NgoAXVI RMS, in each of the 25 *N. gonorrhoeae* strains included in the study. Per-base distribution of IPD ratio is shown as two separate boxplots containing the values in the forward (red) and reverse (blue) strand.

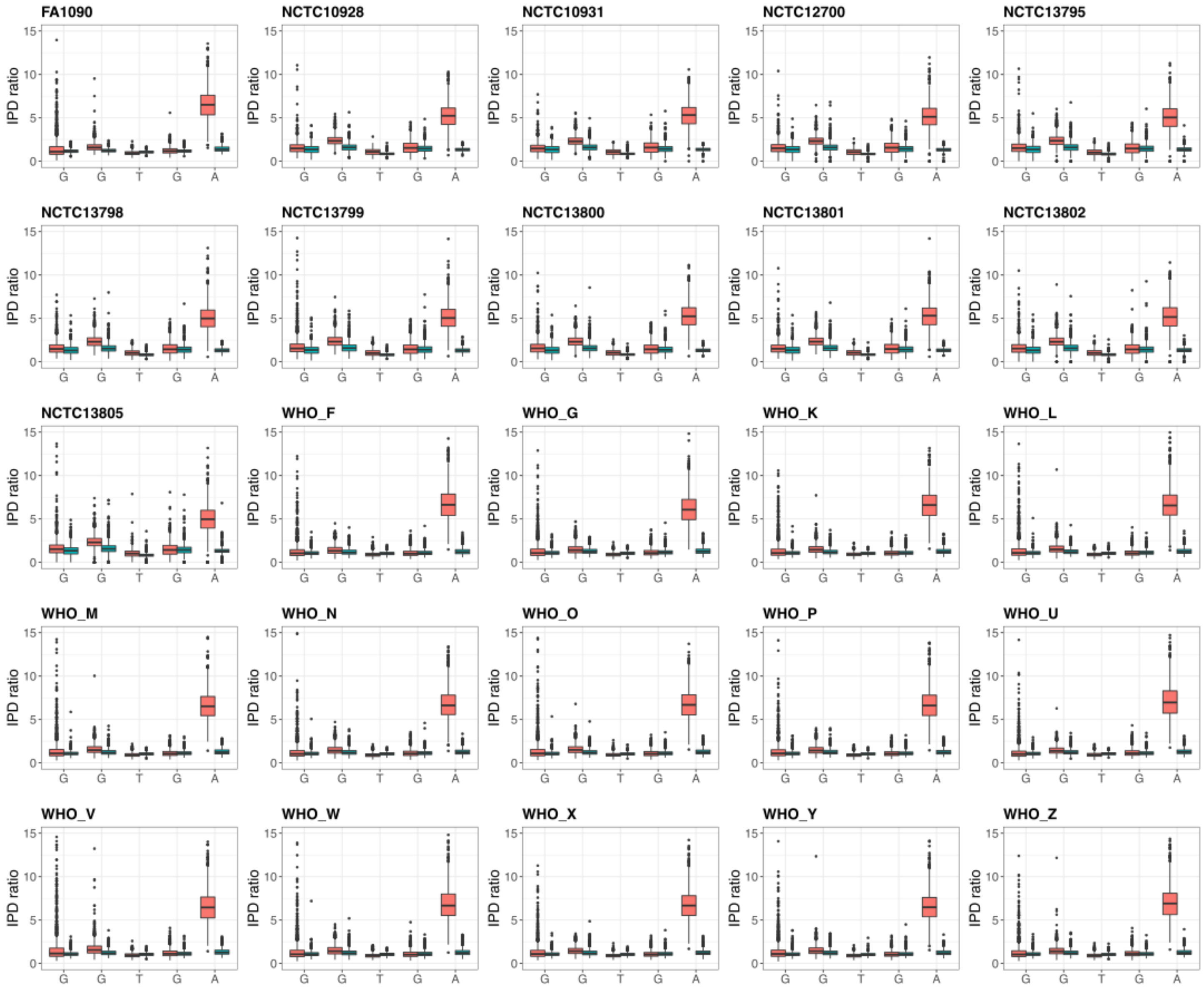

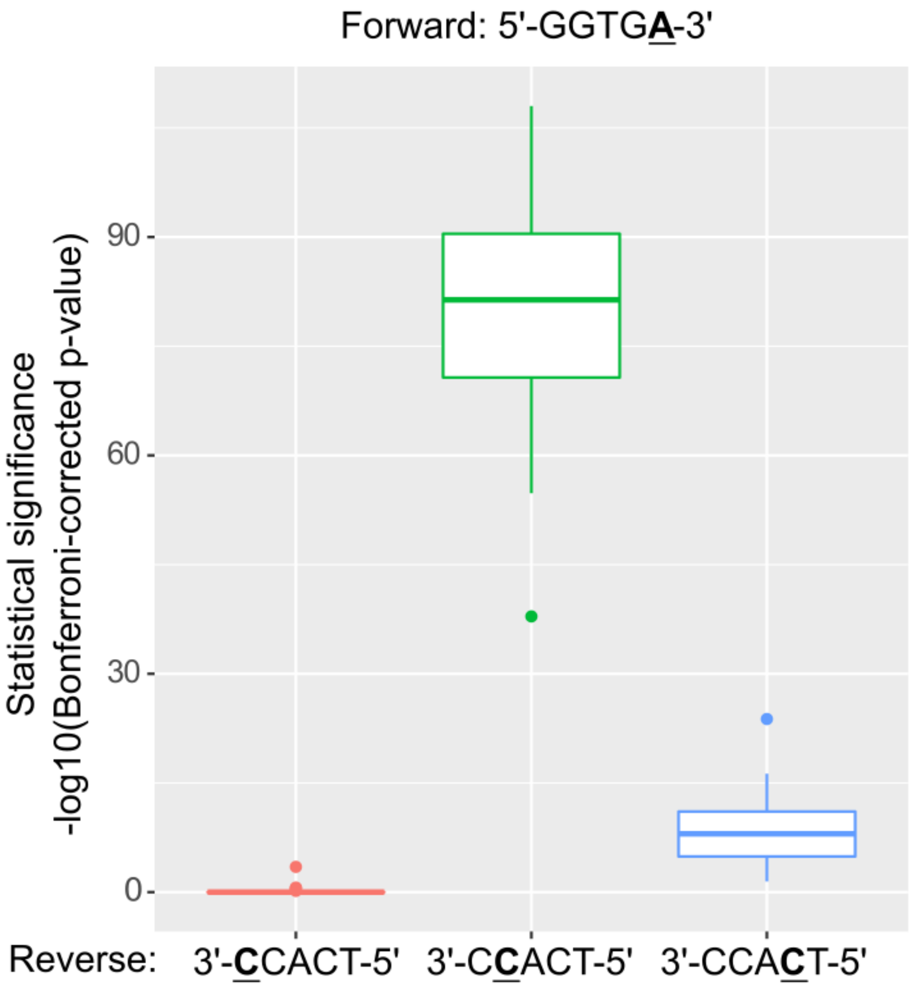

Supplementary Figure 11. Inference of the methylated cytosine in the reverse strand of 5'-GGTGA-3' (NgoAXVI). Bonferroni-corrected p-values are shown in logarithmic scale for the comparison between the distribution of IPD values for the underlined cytosine and a random 10k subsampling of unmethylated cytosines for each strain. The cytosine with a higher significance and thus, candidate to be methylated, is the second one.

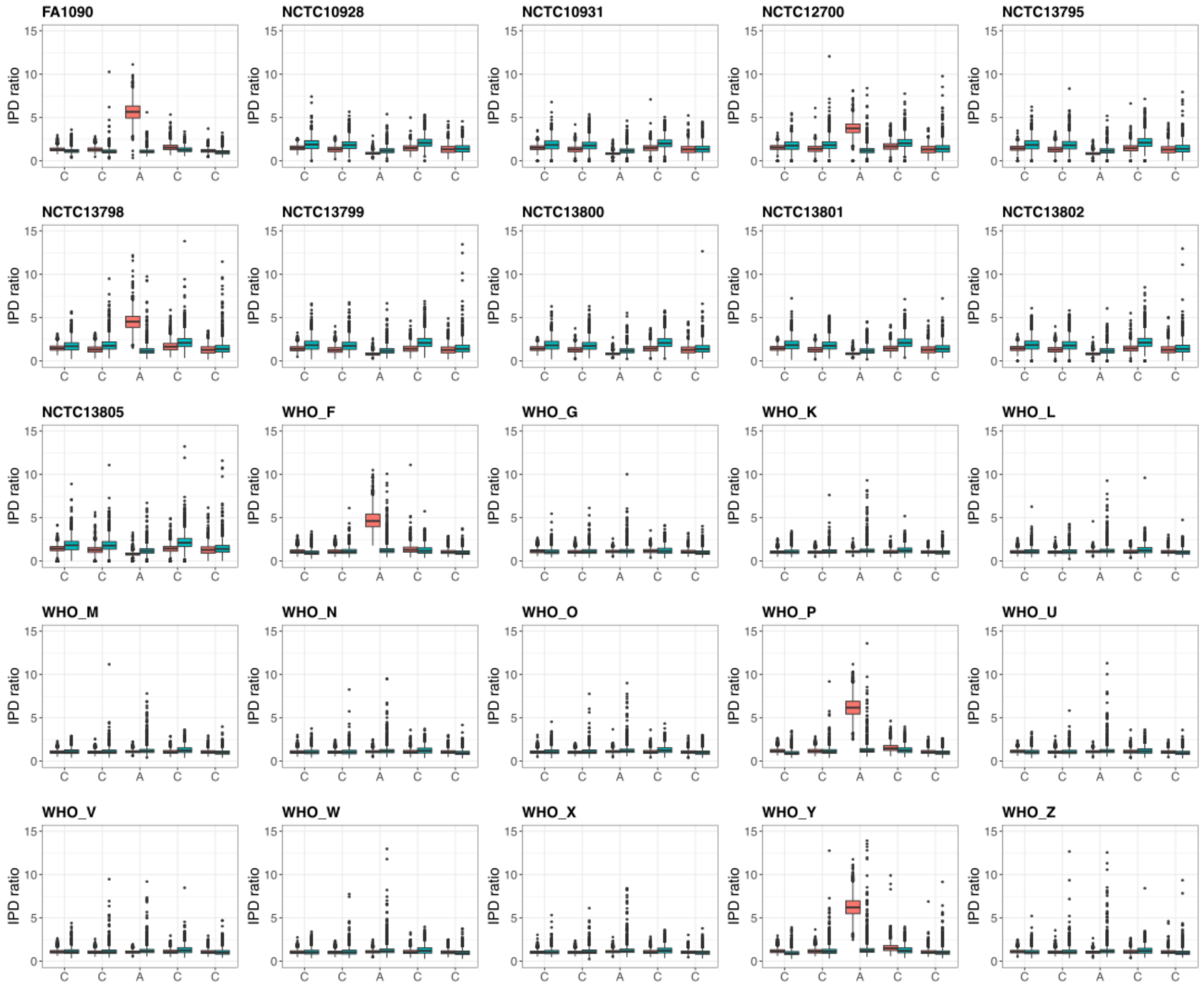

Supplementary Figure 12. IPD ratio values for each base in all instances of the 5'-CCACC-3' motif, target of the Type III NgoAX RMS, in each of the 25 *N. gonorrhoeae* strains included in the study. Per-base distribution of IPD ratio is shown as two separate boxplots containing the values in the forward (red) and reverse (blue) strand.

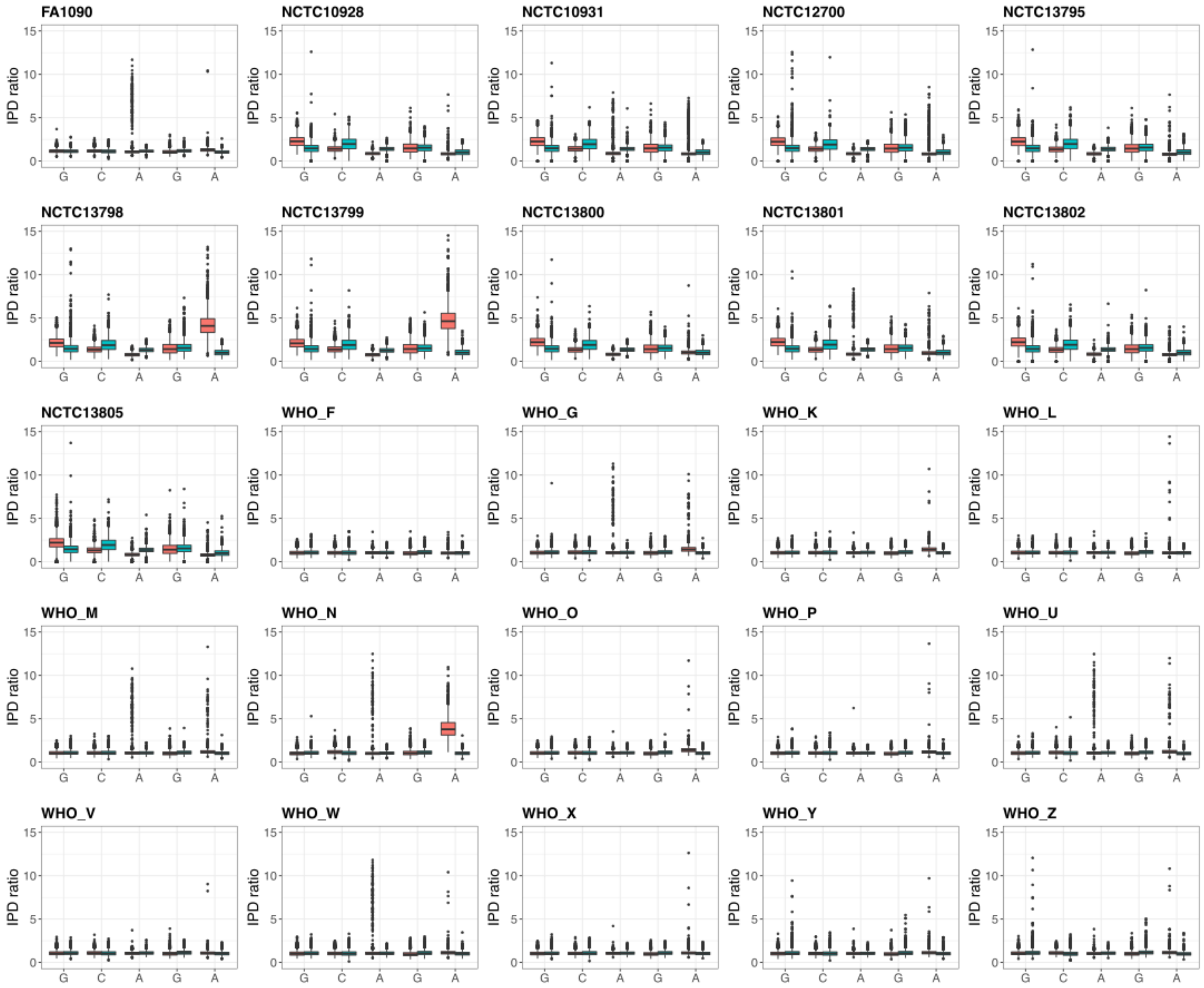

Supplementary Figure 13. IPD ratio values for each base in all instances of the 5'-GCAGA-3' motif, target of the Type III NgoAXII RMS, in each of the 25 *N. gonorrhoeae* strains included in the study. Per-base distribution of IPD ratio is shown as two separate boxplots containing the values in the forward (red) and reverse (blue) strand.

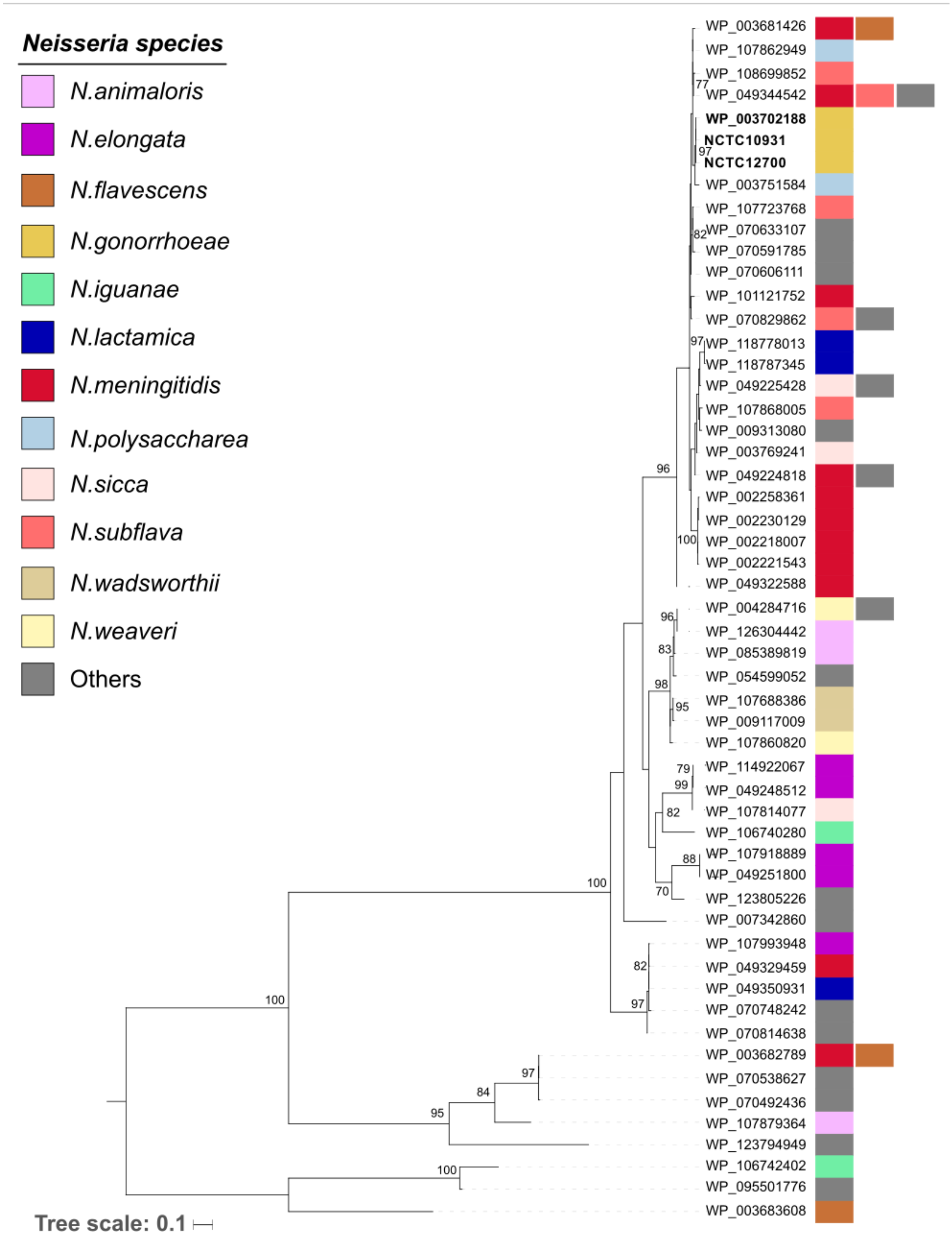

Supplementary Figure 14. Maximum likelihood phylogenetic reconstruction of representative Dam protein sequences available in genomes of the *Neisseria* genus in the RefSeq database. These representative sequences (WP_*) were obtained using the Identical Protein Groups (IPG) tool in NCBI. Coloured strips represent the species in which these sequences are found. Those appearing only once have been coloured in grey as 'Others' but can be accessed using the ID on the tip labels in https://www.ncbi.nlm.nih.gov/ipg.

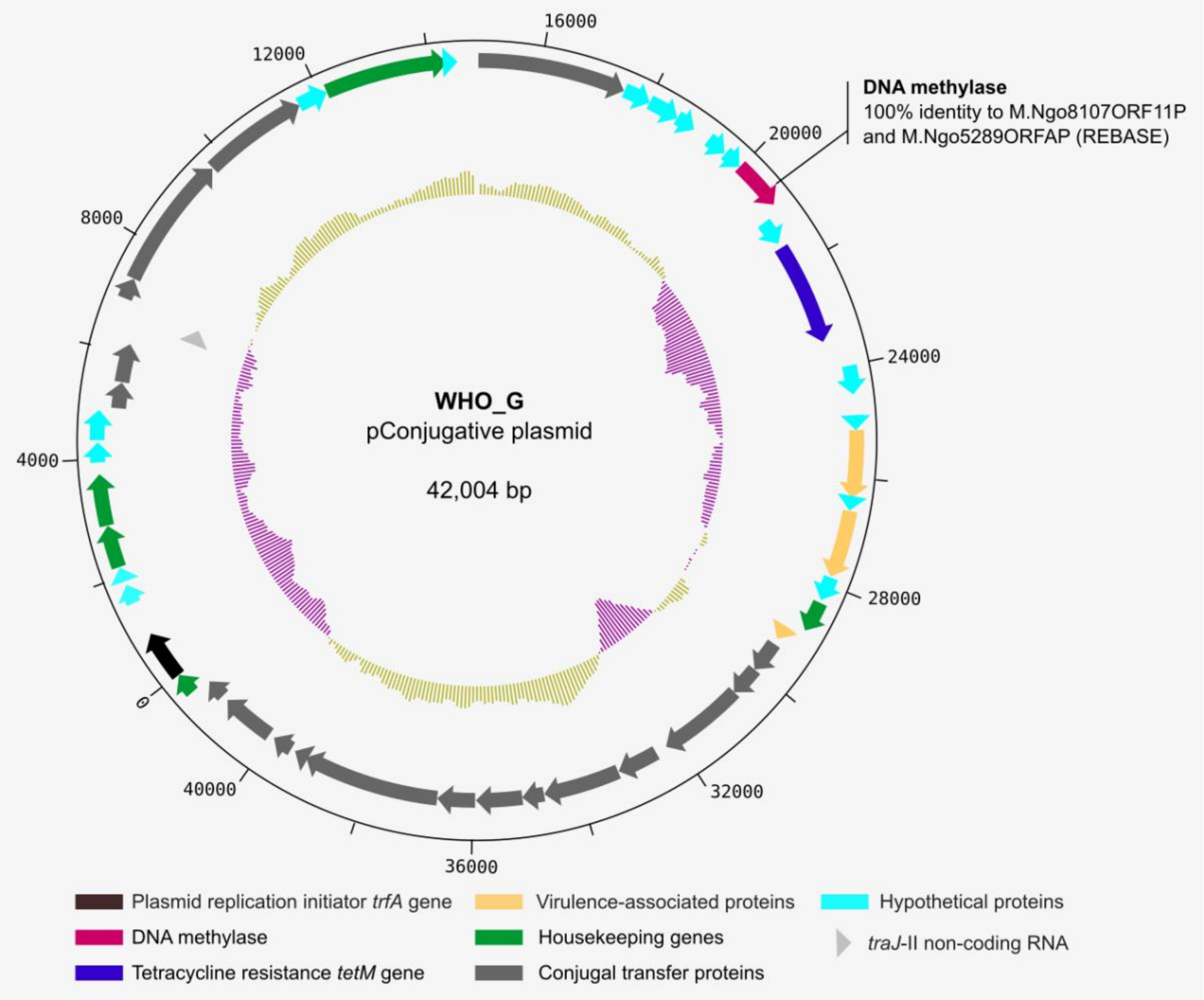

Supplementary Figure 15. Circular plot[58] representing the annotation of the WHO_G pConjugative plasmid, which shows the presence of an orphan DNA methylase that has a 100% protein identity with two entries in REBASE.

58. Carver, T., Thomson, N., Bleasby, A., Berriman, M. & Parkhill, J. DNAPlotter: circular and linear interactive genome visualization. Bioinformatics 25, 119-120, doi:10.1093/bioinformatics/btn578 (2009).
